# Modeling Wetland Resources for Spring Migratory Waterbirds Under Different Agricultural Management Scenarios in the Iowa Portion of the Prairie Pothole Region, USA

**DOI:** 10.1007/s13157-025-01930-y

**Published:** 2025-04-30

**Authors:** Mark E. Mitchell, Michael J. Anteau, Aaron T. Pearse, Tammy Newcomer-Johnson, Jay Christensen, William Crumpton, Brian Dyson, Timothy J. Canfield, Matthew Helmers, David Green, Kenneth J. Forshay

**Affiliations:** 1 Oak Ridge Institute for Science and Education (ORISE) Research Participation Program, U.S. Environmental Protection Agency, 26 W. Martin Luther King Drive, Cincinnati, OH 45268, USA; 2 Biology Department, Xavier University, 3800 Victory Parkway, Cincinnati, OH 45207, USA; 3 Northern Prairie Wildlife Research Center, U.S. Geological Survey, 8711 37 th Street SE, Jamestown, ND 58401, USA; 4 Office of Research and Development, U.S. Environmental Protection Agency, 26 W. Martin Luther King Drive, Cincinnati, OH 45268, USA; 5 Department of Ecology, Evolution, and Organismal Biology, Iowa State University, 251 Bessey Hall, 2200 Osborn Dr., Ames, IA 50011, USA; 6 Office of Research and Development, U.S. Environmental Protection Agency, 919 Kerr Research Drive, Ada, OK 74820, USA; 7 Department of Agricultural and Biosystems Engineering, Iowa State University, 4354 Elings, 605 Bissell Rd., Ames, IA 50011, USA

**Keywords:** Tile Drainage, Ducks, Shorebirds, Constructed Wetlands, Drainage Modernization

## Abstract

Constructed water quality wetlands, designed to accept tile drainage and surface runoff, are a promising solution for reducing surface water nutrient loading from agricultural systems. In addition to their water quality benefits, these systems may also offset losses of migratory waterbird stopover sites resulting from historical and future agricultural drainage modernization. To assess this possibility, we developed spatially explicit habitat models informed with expert opinion to explore the: 1) potential of water quality wetlands to provide suitable stopover resources for waterbirds during spring migration; and 2) the extent these wetlands can offset likely losses of stopover resources due to drainage modernization. We focused our modeling on the Iowa portion of the Prairie Pothole Region of North America as it was a historically important area within this vital region for waterbirds, but it has experienced widespread subsurface drainage. Model results indicate that unmitigated drainage modernization is likely to have a large negative effect on spring migratory resources for dabbling ducks and shorebirds and minimal effect on diving ducks. Water quality wetland installations are likely to provide habitat for dabbling and diving ducks, but wetland installation is unlikely to completely offset habitat losses for dabbling ducks and shorebirds. Drainage modernization aside, our results indicate that water quality wetlands can address several environmental issues associated with agricultural expansion and intensification by improving water quality and providing wetland resources for waterbirds and other organisms. Field-scale research is needed to validate these results.

## Introduction

The Prairie Pothole Region (PPR) in the northcentral United States and southcentral Canada remains the most productive breeding area for ducks in North America (Batt et al. 1989; Doherty et al. 2018; Zimpfer et al. 2012) and a critically important stopover area for migrating duck and shorebird populations (Anteau 2012; Anteau and Afton 2009a; Ballard et al. 2021; Steen et al. 2018). Expansion and intensification of agriculture in the PPR continues to result in widespread wetland drainage and loss of native grassland (Alemu et al. 2020; Dahl 1990; Gallant et al. 2011; Lark et al. 2015) as well as nitrogen export from farm fields via subsurface drainage pipes, hereafter referred to as tile drainage. Nitrogen loss and transport to surface waters contribute to eutrophication and harmful algal blooms locally and hundreds of kilometers away through river systems (David et al. 2010; Mitsch et al. 2001; Rabalais et al. 2002; Schilling et al. 2020). As land managers and farmers seek improved practices that prioritize water quality benefits while maintaining or enhancing crop production (Mitchell et al. 2022b), it is timely to consider the effects of these impending landscape decisions on the duck and shorebird populations that are dependent on this region.

Migratory stopover sites (Lagrange and Dinsmore 1989) provide resources for waterfowl and shorebirds during the spring and are important for fueling flights to sometimes distant breeding habitat and subsequent reproductive success (Anteau and Afton 2004, 2009a; Baker et al. 2004; Krapu et al. 2006; Linscott and Senner 2021; Newton 2006; Stafford et al. 2014). Each species of migratory duck and shorebird uses a different array of wetland resources, with many of them relying on a combination of shorter- and longer-hydroperiod wetlands (Baldassarre et al. 2006; Dwyer et al. 1979; Krapu 1974a), but all seek surface water for food or roost habitat (Kirby et al. 2008). Extant wetlands and flooded areas are critical spring stopover resources for migrating ducks and shorebirds, even those that are farmed and drained but hold surface water for some period during spring (Ballard et al. 2021; Murphy and Dinsmore 2018b). However, the abundance and quality of extant wetlands in intensively farmed landscapes, such as in Iowa, likely is limiting for many of these bird populations (e.g., Anteau and Afton 2011).

**Fig. 1:**
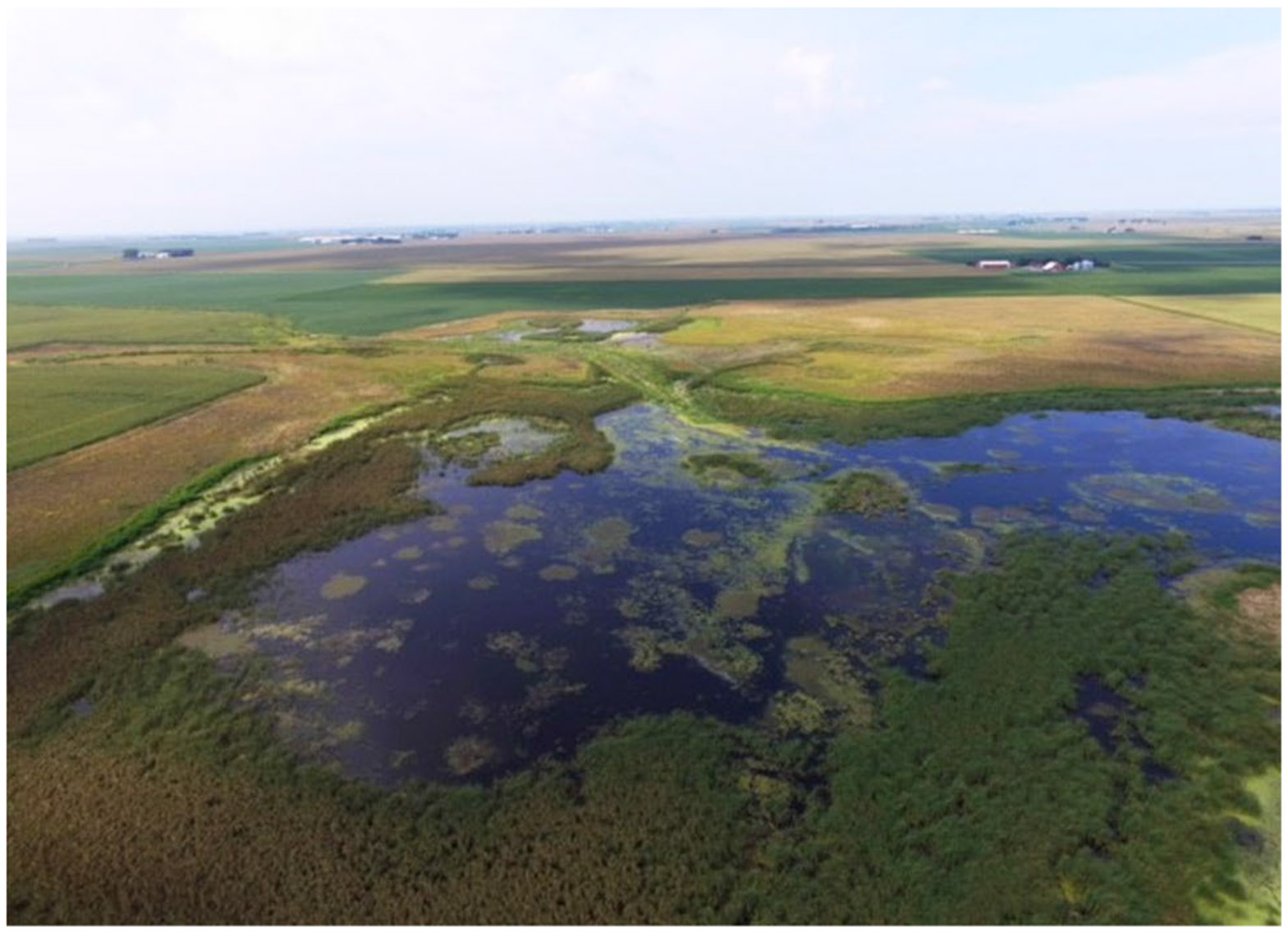
Example of a water quality wetland installed in the Des Moines Lobe of Iowa, courtesy of U.S. Environmental Protection Agency (EPA) Region 7, used with permission

Land management solutions intended to reduce nitrogen export from agricultural watersheds, particularly those solutions that directly or indirectly contribute aquatic resources, may benefit waterbirds in the PPR. Constructed wetlands, hereafter referred to as water quality wetlands (e.g., Fig. 1), are designed to intercept tile drainage and surface runoff, and represent a promising solution that can improve water quality and provide habitat benefits for several organisms (Crumpton et al. 2020; Mitchell et al. 2023). Water quality wetlands and the emerging practice of drainage water recycling are the only existing management practices appropriate for improving subsurface tile drain water quality that directly provide relatively large surface water resources of potential use for waterbirds during spring migration (Ballard et al. 2021; Hay et al. 2021; Mitchell et al. 2022b). We posit that water quality wetlands that functionally resemble natural semipermanent wetlands also could provide stopover resources for certain groups of birds that depend on longer-hydroperiod wetlands. To our knowledge, the potential spring migratory waterbird resources provided by water quality wetlands have not been documented (Mitchell et al. 2022b). Likewise, the ability of water quality wetlands to offset losses of waterbird migratory stopover areas in farmed depressions due to drainage modernization has also not been explored (Mitchell et al. 2022b).

Farmed depressions can pond water for periods of time ranging from days to months due to underperforming drainage infrastructure, and represent resources for migrating waterbirds (Ballard et al. 2021; Murphy and Dinsmore 2018b; Toy 2022) but challenges for farmers (Mitchell et al. 2022b). This is especially the case in the Iowa portion of the PPR where subsurface drainage that was installed in the late 1800 s and early 1900 s is prevalent (Helmers et al. 2009; Hollander 1968; Kanwar et al. 1983). For example, Schilling et al. (2019) modeled ponding in a drained and cropped depression in the Iowa portion of the PPR and estimated that the depression has ponded water 14% to 47% of the time with maximum ponding depth between 112 and 334 mm. Ponded water in cropped depressions can result in crop losses and challenging land preparation for planting (Helmers et al. 2009; Kanwar et al. 1984; Toy 2022). Therefore, many farmers in the Iowa portion of the PPR plan to modernize their drainage systems by replacing and upgrading the tile drainage system laterals and mains which collect drainage from the field tiles and currently operate as bottlenecks (Mitchell et al. 2022b). If executed, such plans might pay off financially for farmers but could result in a loss of habitat for migrating waterbirds during spring.

Herein we employ spatially explicit habitat models informed with expert opinion to explore the potential of a nitrogen management strategy that includes water quality wetlands to provide suitable stopover and refueling resources for ducks and shorebirds during spring migration. We also investigated a scenario where agricultural subsurface drainage modernization would result in a loss of extant shorter-hydroperiod wetland resources and a scenario to investigate if the addition of water quality wetlands could offset these losses associated with drainage modernization. We focus our modeling on the Iowa portion of the PPR, an area of historical importance for ducks and shorebirds (Low 1941) that requires conservation and restoration attention to provide important spring stopover habitat (Anteau and Afton 2009a, 2011). The Iowa portion of the PPR also serves as an example where widespread surface and subsurface drainage have extensively modified the landscape. Therefore, our models may be relevant to assess future agricultural expansion and intensification decisions in other portions of the PPR and in other regions facing similar agricultural and wildlife challenges.

## Methods

### Study Area

**Fig. 2:**
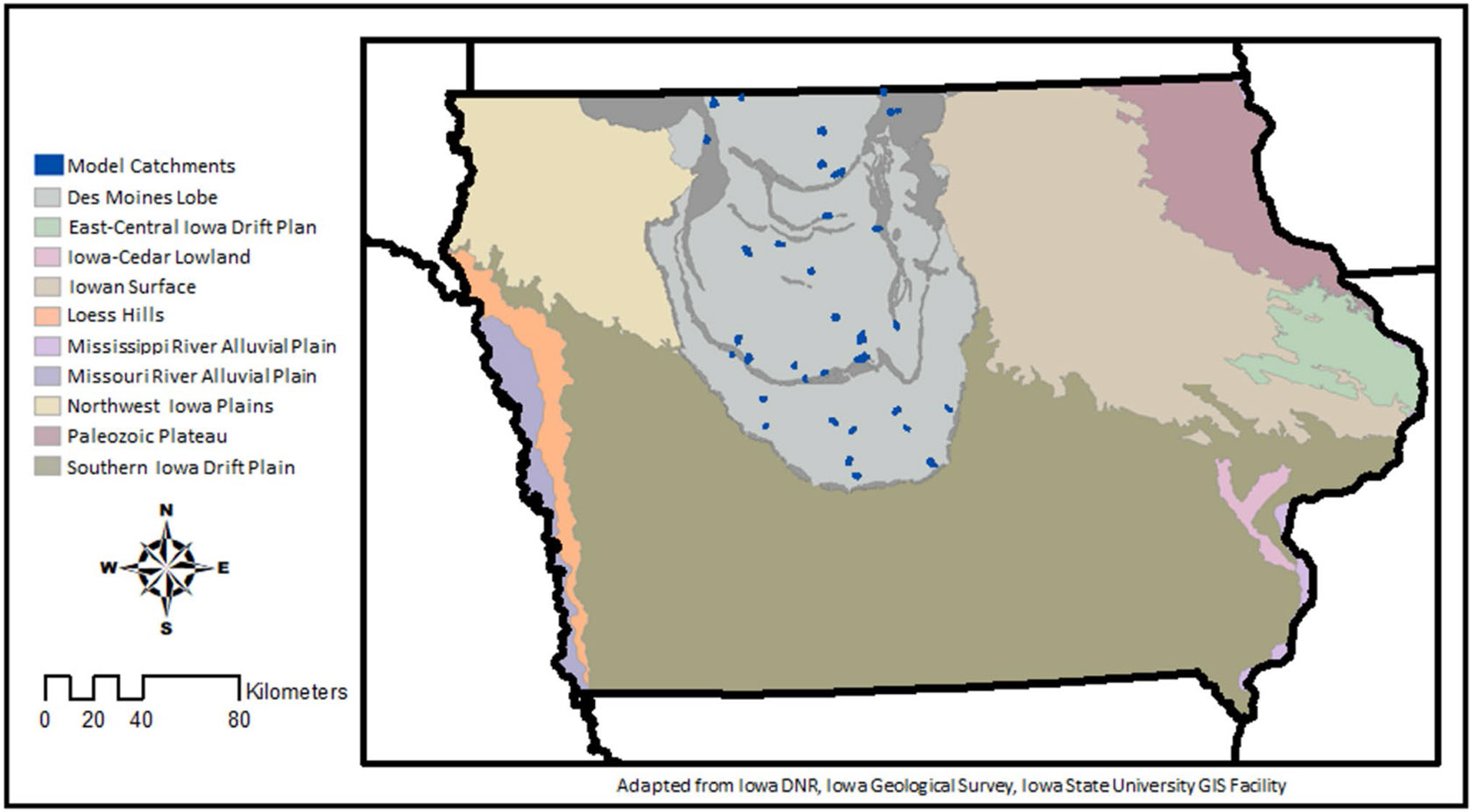
Model catchment locations within the Des Moines Lobe of Iowa, the southernmost portion of the Prairie Pothole Region. Adapted from Mitchell et al. 2022a

The Des Moines Lobe of Iowa encompasses the southernmost portion of the Prairie Pothole Region (Fig. 2) that, before agricultural expansion, was comprised of prairie pothole wetlands within a grassland habitat matrix (Dahl 1990; Gallant et al. 2011). In the mid- 1800 s to early 1900 s, much of the landscape was converted to crops with the installation of subsurface drainage systems made up of ceramic pipes called “tiles” following soon after. There are now more than 173,000 drained depressions in the Des Moines Lobe of Iowa (McDeid et al. 2019). While much of the original ceramic tiles in agricultural fields in the region have now been replaced by perforated plastic pipes which in some cases include surface intakes to effectively drain depressional areas, most of the original large-diameter ceramic pipes that collect drainage from the field tiles remain in use throughout Iowa and are under-designed for the current extent of drainage (Helmers et al. 2009; Hollander 1968; Kanwar et al. 1983; Mitchell et al. 2022b). Widespread drainage modernization, which entails replacing the original ceramic tiles called laterals and mains that receive drainage water from fields, is planned in the region to improve crop yields and field access (Mitchell et al. 2022b).

An emerging practice in the Des Moines Lobe of Iowa is the use of water quality wetlands (e.g., Fig. 1), which are restored or constructed wetlands developed with the primary goal of capturing and treating nutrients in agricultural tiledrainage. These constructed wetlands operate at the small watershed scale and are typically installed at the tile drainage system outlet so that they collect tile drainage and surface runoff from the watershed where they are installed. If the water is held long enough in the water quality wetland, nitrate-nitrogen is removed by microbes through the natural process of denitrification, effectively treating the water with a nature-based solution. Many of the existing systems were installed as a part of the Conservation Reserve Enhancement Program (CREP) but today funding comes from many sources. The average existing CREP wetland pool in Iowa represents 0.85% of the watershed area and the main depth requirement for these CREP style wetlands in Iowa is that 75% of the wetland pool must be three feet or less in depth (Iowa Department of Agriculture and Land Stewardship, written communication, 2022; Mitchell et al. 2022b).

### Model Locations

Watersheds distributed throughout the Des Moines Lobe of Iowa (Fig. 2) were selected from each of the region’s glacial advances and represent a range of depressional water storage capacities within each watershed, as identified by McDeid et al. (2019). ArcGIS Pro 2.8 (Esri, Redlands, CA, USA) was used to identify watersheds and potential locations for water quality wetlands. Three-meter LiDAR-derived contours with 0.6 m elevation intervals were used to delineate watershed and water quality wetland pool boundaries and were obtained from the State of Iowa (https://ortho.gis.iastate.edu/#MapLayers). Thirty-seven watersheds in total were selected for this study that were representative of existing CREP water quality wetlands and their watersheds, with watersheds varying in size from 129 to 1,296 ha and water quality wetland-to-watershed areas ranging from 0.4 to 6.4% (Table 1). Drained depressional area within each watershed, representing depressional areas with cropland and hydric soils as identified by McDeid et al. (2019), ranged from 0 to 20% of the watershed area, with individual depressions having a median area of 0.47 ha and ranging from 0.04 ha to 50.15 ha. As is typical for CREP wetlands in Iowa, water quality wetlands were surrounded by a conservation easement that included at minimum all surrounding areas within 1.2 m elevation above the elevation of the wetland pool. For more specifics about model site selection, refer to Mitchell et al. (2022a).

### Modeled Organisms

Models were produced for individual groups of waterbirds that share similar migratory resources and phenology in the region of interest based on expert opinion. These groups included 1) field foraging dabbling ducks, 2) obligate wetland-feeding dabbling ducks, 3) diving ducks, and 4) shorebirds. For the full list of species considered in each group, see Table 2. All ducks were assumed to reach peak migratory numbers in the region in March and April of each year, whereas shorebirds were assumed to reach peak migratory numbers in the region in April and May of each year (eBird 2021; Kenne 2005; Murphy and Dinsmore 2018a).

### InVEST Habitat Quality Models

**Fig. 3:**
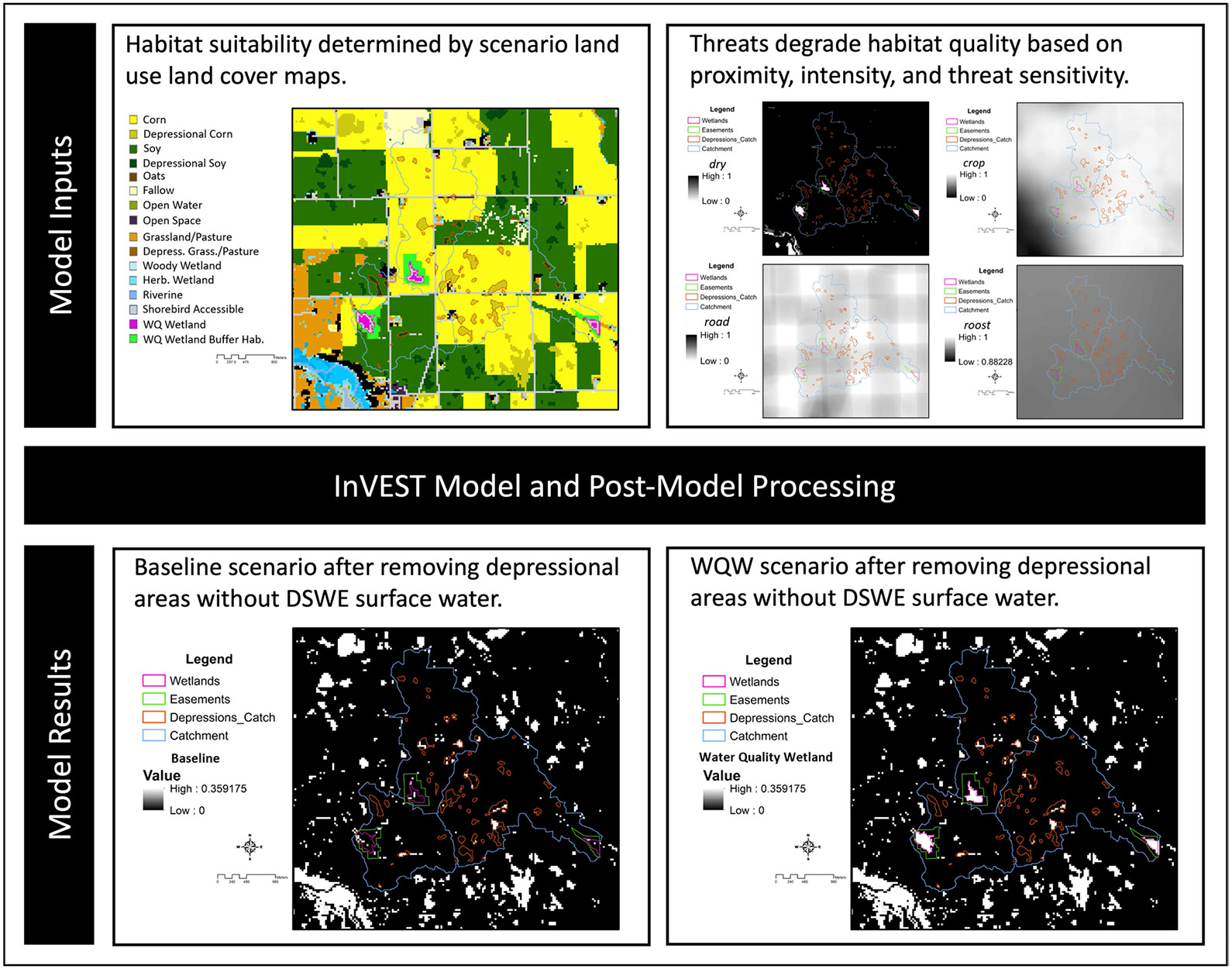
Waterbird modeling process example showing inputs to the Integrated Valuation of Ecosystem Services and Tradeoffs (InVEST) model and results after post-model processing. This process was performed for all scenarios (Baseline, Drainage Modernization, Water Quality Wetland Installation, and Drainage Modernization with Water Quality Wetland Installation) and all modeled waterbird groups in the Iowa portion of the Prairie Pothole Region, USA. Inputs and model results are shown as examples and do not necessarily include all components. WQW = Water quality wetland; DSWE = Dynamic Surface Water Extent; Depress. = Depressional

The Integrated Valuation of Ecosystem Services and Tradeoffs (InVEST; Natural Capital Project) Habitat Quality Model (Sharp et al. 2016) is a spatially explicit model used in this study to estimate habitat and resource implications of land use scenarios on different groups of waterbirds. Spatial layers with land use and land cover information were paired with expert opinion to identify the maximum potential habitat quality, termed habitat suitability (see Sect. 2.4.2), in a particular location for each waterbird group of interest (Fig. 3). The Habitat Quality model then degrades these habitat suitability scores based on the location and proximity of threats. Threats (see Sect. 2.4.3) are considered environmental factors that can disrupt habitat quality and include short hydroperiods, agricultural production, or the location of disturbances such as roads (Fig. 3).

Habitat Quality, a numerical index, is calculated based on habitat suitability as determined by expert opinion, threat proximity, the relative influence of threats on habitat quality (threat weights), and the relative sensitivity of a specific habitat type to a specific threat. Habitat quality degradation due to threats is additive across all applicable threats. The habitat quality index calculation is a half-saturation function and the user-specified half-saturation constant of 0.5, after Mushet and Roth (2020), was used for all models. The habitat quality index, Q, for a given map cell x in land use type j, is calculated with the following equation:

$$Q_{xj}=H_{j}(1-(\frac{D_{xj}^{z}}{D_{xj}^{z}+k^{z}}))$$

where $H_{j}$ is the habitat suitability in land use type j, $D_{xj}$ is the combined threat level in map cell x with land use type j and affected by threat proximity and the sensitivity of land use type j to threats, z is set to a value of 2.5 as the scaling constant, and k is set to 0.5 as the half-saturation constant. For more specifics and calculations, please see the sections below and Mitchell et al. (2022a) and Sharp et al. (2016).

#### Scenario Land Use Land Cover Maps

Land use and land cover information is necessary for the InVEST model as it informs the location of potentially suitable habitat and provides spatially explicit information about the presence of co-located or surrounding threats to potential habitat locations that are used to calculate a habitat quality score (Sharp et al. 2016). Land use land cover (LULC) maps with a resolution of 30 by 30 m were generated for the region of interest by overlaying the 2019 National Agricultural Statistics Survey Cropland Data Layer (CDL; U.S. Department of Agriculture National Agricultural Statistics Service, Cropland Data Layer Accessed August 18, 2021. https://www.nass.usda.gov/Research_and_Science/Cropland/SARS1a.php) drained depressions layer (McDeid et al. 2019), and National Wetlands Inventory (NWI; U.S. Fish and Wildlife Service, National Wetlands Inventory; Accessed August 8, 2020. https://www.fws.gov/wetlands/) using the methods provided for modeling amphibians in Mitchell et al. (2022a) and highlighted in Fig. 3. Briefly, drained depressions were delineated by McDeid et al. (2019) as locations with cropland and hydric soils. Drained depressions were further identified based on the majority LULC in the 2019 CDL occurring within these locations. Additionally, the location of roadways was overlaid on this base map (Tiger/Line city census data; U.S. Census Bureau; Accessed November 4, 2020; www.census.gov/geographies/mapping-files/time-series/geo/tiger-line-file.2019.html). All LULC geographic information systems analyses were performed in ArcMap 10.8.1 (Esri, Redlands, CA, USA). For more specifics about LULC map methods, see Mitchell et al. (2022a).

The following scenarios, each with its own scenario LULC layer for InVEST modeling, were evaluated for effect on habitat quality: baseline (no change); drainage modernization; drainage modernization with water quality wetlands; and water quality wetlands without drainage modernization.

The baseline map consisted of the base land-cover map as described previously, which included drained depressions, NWI wetland locations, NWI lacustrine littoral edge locations with a negative (interior) buffer distance of 30 m, and CDL LULC information.

The drainage modernization scenario employed a worstcase scenario approach and assumed the complete loss of any aquatic resources occurring within drained depressions within model watersheds in the spring, with drained depression locations converting to the non-depressional LULC type based on the majority LULC in the 2019 CDL (e.g., depressional corn converted to corn). Any wetlands occurring within depressions as identified by NWI or CDL were also converted to the majority LULC in the depression from the 2019 CDL in this drainage modernization scenario.

Scenarios with water quality wetland additions involved overlaying modeled water quality wetland locations with surrounding habitat buffer locations (Fig. 3). These modeled water quality and buffer locations were representative of existing Iowa CREP water quality wetland locations as described previously in Sect. 2.2 and Table 1. Modeled water quality wetland locations were overlaid on top of the baseline land cover map in the water quality wetland scenario without drainage modernization while water quality wetland locations were overlaid on the drainage modernization land cover map in the drainage modernization with water quality wetland scenario. In water quality wetland scenarios, an additional layer was mosaiced on top of the water quality wetland layer to delineate areas potentially accessible to shorebirds in April–May, defined as depths of 20 cm or less based on expert opinion (Fig. 3). This shorebird accessible shoreline was characterized in ArcMap using a negative (interior) buffer of one pixel (30 m, the minimum resolution). Models without this accessible shoreline area were also evaluated.

#### Habitat Suitability

For each modeled group of species, suitable habitat was designated using land use land cover classes as described previously and expert opinion. Unsuitable habitat for use by waterbird groups in the spring was given a value of zero, while potentially suitable habitat was given a value of 0.5 or one to identify the maximum potential habitat quality score based on expert opinion of potential food availability (Supplemental Tables 1, 3, 5, 7). A value of one represented the most suitable habitat type under ideal conditions for spring migratory resources. Local conditions likely to affect stopover habitat use such as hydrology, proximity of roost sites, and nearby road disturbance were accounted for later with threats (Fig. 3).

Water quality wetlands in the model were treated as semipermanent wetlands, but were expected to have more submerged aquatic vegetation and less emergent vegetation compared to natural wetlands within the first three years of restoration (Galatowitsch and van der Valk 1996). The longer-term hydroperiod of water quality wetland systems has not been evaluated.

Drained, cropped depressions were overlaid on top of the cropland data layer and identified by McDeid et al. (2019; Fig. 3) and were considered as suitable habitat with a high capacity for food availability for all waterbird groups except for diving ducks based on their tendency to intermittently flood in the spring and provide sheetwater and mudflat habitat with high potential for invertebrate and moist-soil seed availability (Murphy and Dinsmore 2018b; LaGrange 1985). However, drained depressions that rarely had surface water in estimates based on satellite imagery were later downgraded with the application of threats and post-model analyses (Fig. 3; see Sect. 2.4.3). The NWI layer and CDL wetland specific LULCs were added on top of this depressional layer so that NWI designated wetlands could occur within cropped depressions. For more specifics about the depressional layer and the mosaicing process, see Mitchell et al. (2022a).

#### Threats

InVEST uses threats to degrade the habitat quality score based on habitat type and/or the characteristics of nearby LULCs that result in less desirable habitat at a particular location (Fig. 3). The effect of a habitat threat is based on the LULC and its proximity to other LULCs, as well as a predetermined impact weight of the threat. All threats were set to have a linear relationship with distance, such that the effects of the threat on the habitat quality index of a particular location decrease linearly over a prescribed distance (Supplemental Tables 2, 4, 6, 8). Habitat threats included in waterbird models and the distance over which they degrade surrounding habitat were identified by expert opinion and literature review and included: density of cropland within 1 km (*crop*); density of corn within 5 km (*corn*); density of roads within 0.5 km (*road*); density of roost sites within the typical flight distance of each group (*roost*); wetland size for diving ducks (*wetarea*); and wetland permanence (*dry*). Other factors, notably wetland size and density, are recognized as potentially important for dabbling ducks in the region during migration (Ballard et al. 2021; Murphy and Dinsmore 2018a). However, Ballard et al. (2021) observed an approximately linear wetland area-dabbling duck use day relationship, indicating little change in dabbling duck use when normalized by wetland area. Additionally, Ballard et al. (2021) observed an inverse relationship between duck use days and wetland density, likely an effect of habitat rarity and not preference. Thus, wetland density was not accounted for outside of the roost proximity threat, and wetland area was only incorporated as a threat in the diving duck model for which more established relationships exist.

Nearby cropland can simultaneously impair and provide food resources for different waterbird groups. Cropland can affect waterbird presence and use directly due to regular disturbance by field operations in the spring and indirectly through effects that impair food resources. For example, agricultural pesticide runoff, pesticide spray drifts, and other factors may impair macroinvertebrate populations and moist-soil seed availability in agricultural wetlands and depressions, resulting in reduced food availability for waterbirds (Euliss and Mushet 1999; Henry et al. 2020). Therefore, the density of cropland within one km of each pixel was used as the input for the *crop* threat raster. Conversely, dabbling field foraging ducks use crop residues, especially from corn, as feed during spring migration and staging and are more likely to use semipermanent wetlands that have high coverage of corn within five km (Anteau et al. 2011b; Ballard et al. 2021). Therefore, any wetland areas without corn within five km were considered threatened in the dabbling field-foraging duck model. Soybeans, on the other hand, are related to reduced use by ducks in wetland systems, particularly in Iowa farmed wetlands (Ballard et al. 2021; Lagrange and Dinsmore 1989). However, the effects are minimal and were therefore treated as having no beneficial or negative effects beyond the negative effects of cropland discussed previously.

Roads (*roads*) can disturb wetland habitat and result in reduced visits and use. For example, Ballard et al. (2021) identified greater duck use of farmed wetlands that were further from roads. Murphy and Dinsmore (2018a, b) identified distance to road as an important variable for determining sheetwater use (abundance) by all waterbirds. Similarly, Lagrange and Dinsmore (1989) observed distance to road was important for estimating mallard use of sheetwater wetlands, but mallards still regularly used sheetwater wetlands within close proximity to roads. Roads were considered threats when they occurred within 500 m of a potential suitable habitat location based on expert opinion and findings by Pfost (2008). The density of roads within a 500 m radius from each pixel was calculated for each scenario land cover map using focal statistics and used as input for the road threat raster layer.

Besides food and water, migrating waterbirds require access to appropriate roost sites (*roost*) (Erwin 1996; Kirby et al. 2008; Lagrange and Dinsmore 1989; Pearse et al. 2011). All potential habitat LULCs for each group, except depressional LULCs due to their ephemeral nature, were classified as roost locations. During migration stopovers, wetland obligate dabbling ducks were observed to fly a maximum of five km in a review of flight distances by Johnson et al. (2014), and therefore were considered threatened when potential roost habitat occurred at lower densities within this distance in all directions from a given pixel. Similarly, dabbling field-foraging ducks with a low density of roost habitat within 18.5 km of a potential habitat location as reviewed by Johnson et al. (2014) were considered threatened. During migration stopovers, lesser scaup, a diving duck, moved a minimum of four km each day on average (A.D. Afton, Louisiana State University, unpublished data), and therefore locations were threatened when there was low availability of roost sites within this flight distance. Shorebirds were identified as having a maximum movement distance of 10 km during migration stopovers, based on studies of pectoral sandpipers (Farmer and Parent 1997) and semipalmated sandpipers (Neima et al. 2020; Obernuefemann et al. 2013), with locations having a low density of relevant roost LULCs within this 10 km treated as threatened.

Wetland area (*wetarea*) is correlated to depth and volume in the Prairie Pothole Region (Minke et al. 2010), and thus an important factor for diving duck use. Based on expert opinion, wetlands with areas greater than four hectares (10 acres) received no threat as this is the minimum size for scaup (Anteau 2006; Anteau and Afton 2009b), wetlands less than four hectares but greater than or equal to 0.4 hectares (one to nine acres) received an intermediate threat level of 0.5, and wetlands smaller than 0.4 hectares (one acre) received the highest threat level of 1.0. Wetland areas in the region of interest were determined using the Region Group tool in ArcMap with a setting of eight neighbors and the Within Zone grouping method.

Wetland permanence (*dry*) potentially affects waterbird use of stopover habitat in several ways. Wetlands or drained depressions that have very short hydroperiods only contribute to waterbird habitat when they pond water. On the other end of the spectrum, permanent wetlands are likely to sustain fish populations which can impair available food resources needed to fuel migration and subsequent breeding success (Anteau and Afton 2008; Anteau et al. 2011a; Janke et al. 2019). Considering the importance of hydroperiod for supporting stopover habitat, we used remote sensing to estimate the frequency of ponding for depressions and wetlands within our study areas.

Dynamic Surface Water Extent (DSWE; Jones 2019) is a dataset developed to predict the extent of surface water from Landsat. We obtained DSWE products using the USGS Earth Explorer (U.S. Geological Survey. Accessed March 16, 2023. https://earthexplorer.usgs.gov/). We produced two raster layers to estimate: 1) the frequency of surface water extent for March–April, and 2) the frequency of surface water extent for April–May. Based on expert opinion, Murphy and Dinsmore (2018a), Kenne (2005), and eBird (2021), the March–April surface water frequency estimates were used for duck models and April–May surface water frequency estimates were used for the shorebird model. From 2013–2022, there were seven years where DSWE products with 60% or less cloud cover were available for March or April for all model watersheds (2013, 2015, 2016, 2017, 2018, 2019, 2020) and seven years where DSWE products with 60% or less cloud cover were available for April or May for all model watersheds (2013, 2014, 2016, 2017, 2018, 2019, 2021). This method ensures we have at least one DSWE product for the period of interest for all drained depressions within model watersheds in each year. On average, depressions within watersheds were captured in 2.8 DSWE products each year during the March and April migratory period for ducks, with a median of 20 total observations for each depression (Supplemental Fig. 1). For the shorebird migration period (April and May), depressions were captured in 2.8 DSWE products each year on average, with a median of 21 total DSWE observations for each depression (Supplemental Fig. 2).

For each relevant DSWE product, pixels with a value of one (Open Water High Confidence), two (Open Water Moderate Confidence), or three (Partial Surface Water Conservative) were reclassified as water and all other values as no water. March and April reclassified DSWE products were mosaiced together for each year using the maximum value for each pixel where multiple DSWE products overlapped. This operation was repeated for reclassified DSWE products from April and May. March–April and April–May mosaics indicating where surface water was identified in a DSWE product from the respective months of each relevant year were mosaiced together using the sum mosaic operator in ArcMap. This summed raster, herein referred to as the Presence/Absence DSWE summarization method, represented the total number of years a pixel was identified as having high to moderate confidence surface water in March or April, and April or May, and we subtracted the fraction of years where surface water was present out of the seven total years of records from a quantity of one to create the *dry* threat layer. We also ran models using the overall frequency of surface water extent across all available DSWE products within drained depressions, herein referred to as the All Measurements DSWE summarization method. To calculate, we divided the total DSWE observations with surface water for a particular location by the total DSWE observations for that same location using the raster calculator tool in ArcMap, and then subtracted this frequency from a value of one to produce the *dry* threat layer. Both surface water frequency results were also used in model postprocessing (Fig. 3) to filter out depressions with no DSWE surface water extent records, since depressions will generally only provide resources for waterbirds when they pond water.

We also ran a series of models for each waterbird group using a range of different threat weights to better understand the influences of threats and to further bound the potential effects of drainage modernization and water quality wetland additions. All threat raster layers except *dry* and *wetarea* were determined using the Focal Statistics tool in ArcMap and calculated as the density of the threat within a relevant radius based on expert opinion for each pixel value. For *roos*t, *corn*, and *dry*, raster cells were rescored by subtracting the raster cell value from a value of one so that areas with a relatively high density of roost habitat, density of corn, or prevalence of water were given the lowest threat values.

## Results

### Field Foraging Dabbling Duck Habitat

Within our 37 watersheds covering a total of 18,837 ha, potentially suitable habitat for field foraging dabbling ducks amounted to 1,701 ha, or 9% of the total evaluated area, based on expert opinion and our baseline scenario land cover map. Drained depressional areas within the modeled watersheds, which in total accounted for 1,484 ha, represented 85% of the total potentially suitable habitat for dabbling field foragers. Overall, 98% of drained depressional area within the model watersheds was potentially suitable habitat. Based on DSWE analyses for all modeled duck groups, 81% of the depressional area in our modeled watersheds did not have any DSWE observations of surface water in March or April (Supplemental Fig. 3).

The InVEST habitat quality score for the baseline scenario across all modeled watersheds, which accounted for habitat degradation in these potential habitat locations due to threats as described previously, totaled 2,826 for dabbling field foraging ducks. However, this score dropped 71% to just 809 when depressional areas that had no surface water activity in any of the available DSWE products were removed from the analysis.

**Fig. 4:**
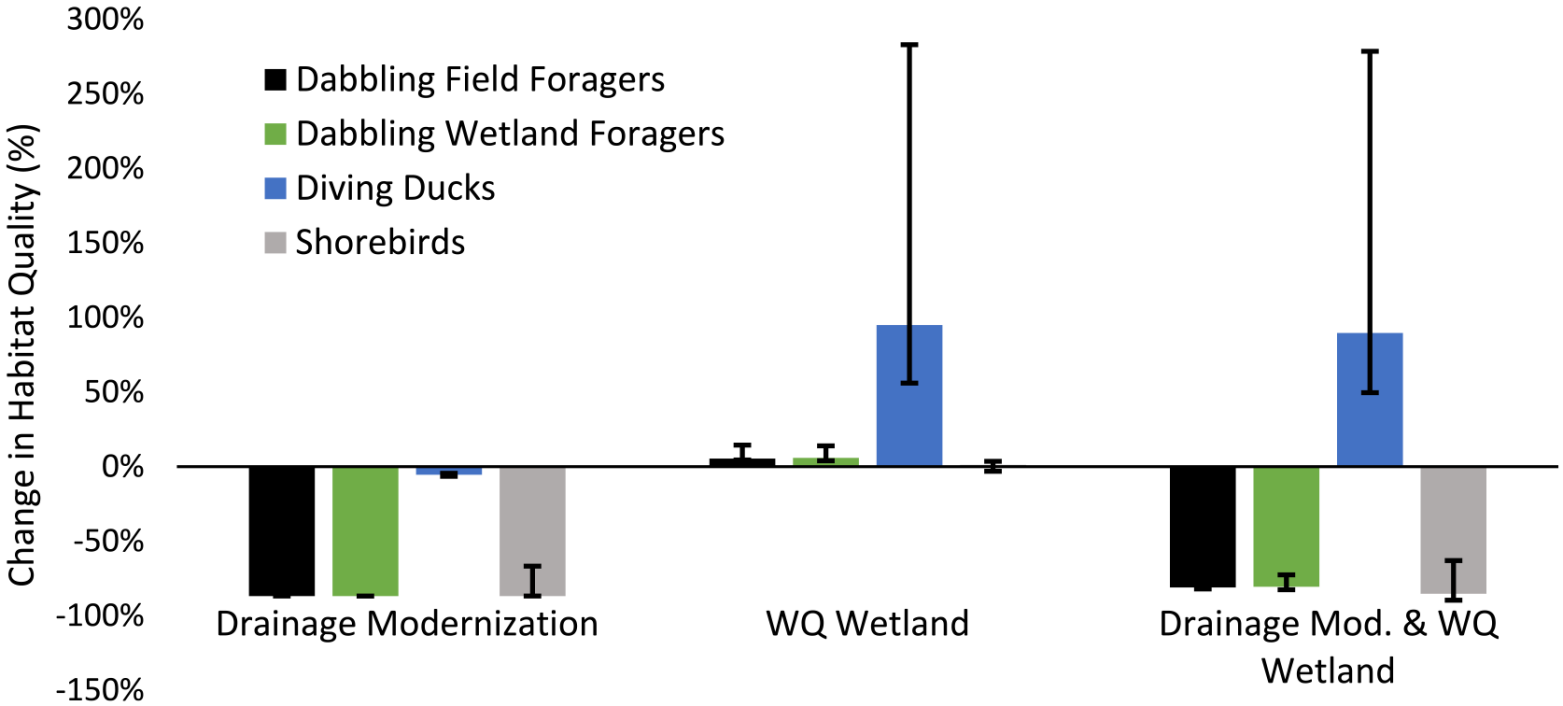
Habitat quality change (%) across all modeled groups in the Iowa portion of the Prairie Pothole Region, USA when all depressional area is considered in the analyses. Results are presented for scenarios with drainage modernization, water quality wetland installation, and both practices combined. Habitat quality change is shown as the percent change relative to the baseline (no landscape change) scenario. Error bars correspond to additional variability identified by testing different model threat weights. For shorebirds, error bars also include variability due to models with and without shoreline habitat

**Fig. 5:**
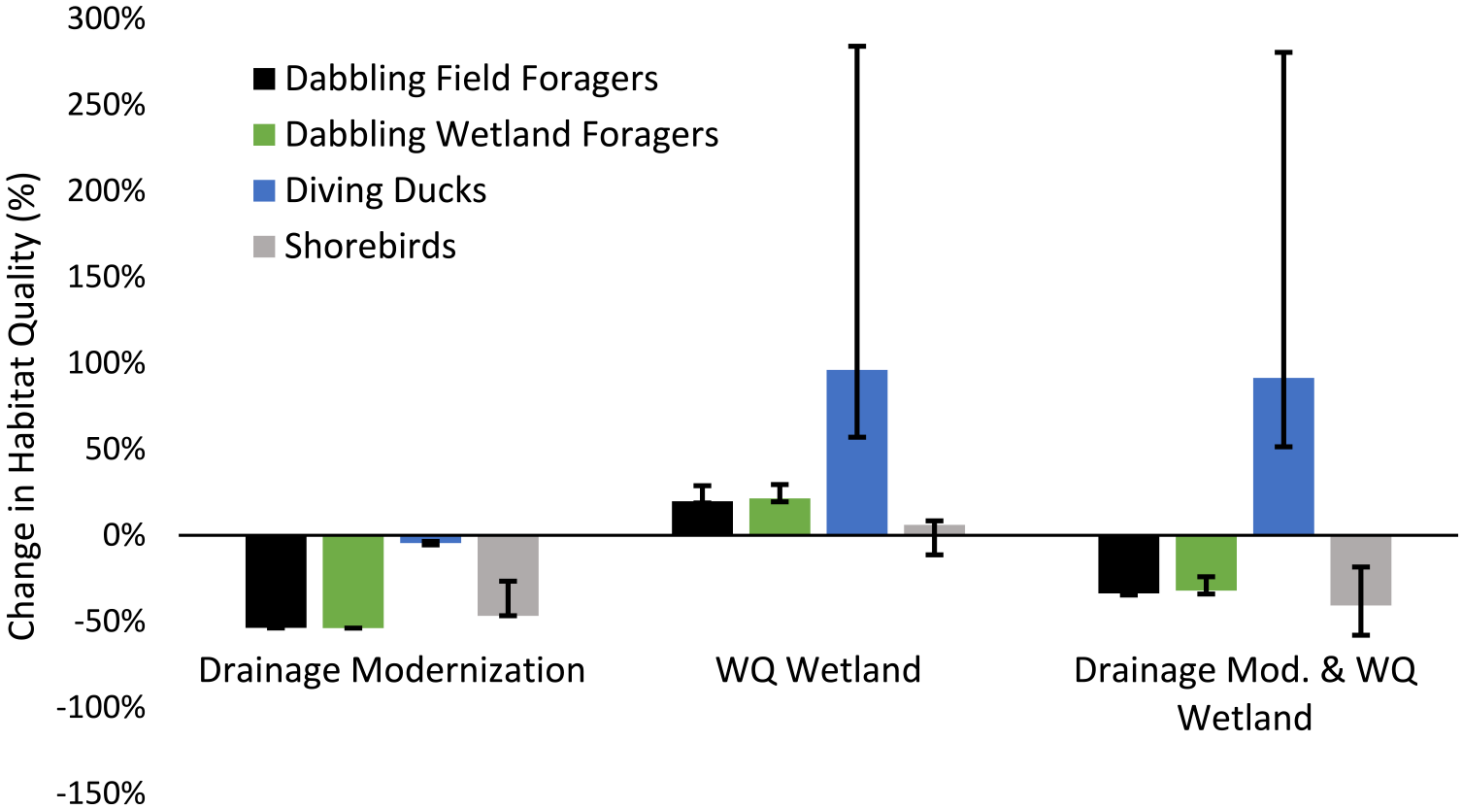
Habitat quality change (%) across all modeled groups in the Iowa portion of the Prairie Pothole Region, USA when depressions without surface water are removed from the analyses. Depressions without surface water are drained depressional areas where surface water was not present in any of the available Dynamic Surface Water Extent (DSWE) products for the spring migratory period. Results are presented for scenarios with drainage modernization, water quality wetland installation, and both practices combined. Habitat quality change is shown as the percent change relative to the baseline (no landscape change) scenario. Error bars correspond to additional variability identified by testing different model threat weights. For shorebirds, error bars also include variability due to models with and without shoreline habitat. Mod. = Modernization; WQ = Water Quality

The drainage modernization scenario resulted in a predicted 87% reduction in the habitat quality score relative to the baseline scenario if all watershed depressional area was considered (Fig. 4). An area totaling 1,439 ha with a relatively high habitat map cell quality score (> 0.15) before drainage modernization was lost or reduced to minimal habitat quality (≤ 0.01) after drainage modernization (Supplemental Fig. 4). When depressional areas within watersheds that were not recognized as having any surface water in DSWE products were removed from the analysis, reductions in habitat quality due to drainage modernization ranged between 54 and 56%, (Fig. 5; Supplemental Table 9).

The scenario with water quality wetland additions to all 37 watersheds resulted in a five percent improvement in the total habitat quality score relative to the baseline scenario (Fig. 4). This included a shift of 174 ha from a low habitat quality score (≤ 0.01) to an intermediate or high habitat quality score (0.05—0.2), and a net 44 ha reduction in relatively high habitat quality (> 0.15) area to an intermediate habitat quality score (0.05—0.15; Supplemental Fig. 4). Combining water quality wetlands with drainage improvements resulted in an 81% reduction in habitat quality if all depressions were considered, ranging down to a 34% reduction if only watershed depressions with DSWE surface water observations were considered (Fig. 5).

Changing the threat weight settings to further bound the effects of drainage modernization and water quality wetland installation indicated minimal additional variability (< 1%) in the habitat quality score from the base model for drainage modernization effects but a − 1% to + 9% variance in the habitat quality score from the base model for water quality wetland additions (Table 3). The *dry* threat weight was the most responsive, improving the habitat quality score by 9% for the water quality wetland scenario when the threat weight was elevated relative to the other threats. Increasing the weight of other threats generated minimal (≤ 1%) change in the habitat quality score for all scenarios. Using different DSWE summarization methods (i.e., annual presence/absence vs. frequency across all measurements) for the *dry* threat and postmodel processing resulted in 2% variance in habitat quality scores (Supplemental Table 9).

The scenarios and sensitivity tests highlighted above bound the effects on field-foraging dabbling duck habitat quality between − 87% and − 54% for drainage modernization and between + 4% and + 29% for water quality wetland additions (Figs. 4 and 5).

### Obligate Wetland-Foraging Dabbling Duck Habitat

Wetland-foraging dabbling ducks, as with the field-foraging dabblers, had 1,701 ha of potentially suitable habitat across all 37 model watersheds, with the majority (85%) occurring in drained depressional areas. After accounting for threats which degraded the habitat quality score in these areas, wetland-foraging dabbling ducks received a total InVEST habitat quality score across all watersheds of 2,840 for the baseline scenario. The habitat quality score declined by 71% to 814, however, when depressional areas with no DSWE surface water observations were removed from the analysis.

Drainage modernization models for obligate wetland-feeding dabbling ducks indicated an 87% reduction in habitat quality relative to the baseline when all depressions regardless of their hydrologic activity were included (Fig. 4; Supplemental Table 9). This reduction amounted to a shift of 1,439 ha of modeled habitat from a relatively high habitat quality score (> 0.15) to a low (< 0.01) or zero habitat quality score (Supplemental Fig. 4). When depressional areas with no DSWE surface water observations throughout the observation period were removed from the analysis, habitat quality declined by 54% to 56% relative to the baseline for the drainage modernization scenario (Fig. 5; Supplemental Table 9).

The water quality wetland additions scenario resulted in an increase in the habitat quality score of six percent relative to the baseline scenario (Fig. 4). Water quality wetland additions resulted in a shift of 174 ha of low habitat quality (≤ 0.01) area to intermediate and high (0.05–0.2) habitat quality, which was accompanied by a net 41 ha reduction in relatively high habitat quality (> 0.15) area (Supplemental Fig. 4). Combining water quality wetlands with drainage modernization resulted in an 81% reduction in habitat quality when all depressions were considered (Fig. 4), ranging down to a 32% reduction in habitat quality if only depressions that were active with surface water at least once were considered (Fig. 5).

Altered threat weight settings for the drainage modernization and water quality wetland addition scenarios indicate minimal (< 1%) additional variability for the drainage modernization scenario but an additional variance of − 2% to + 8% to fully bound the potential effects of water quality wetland additions (Table 3). As with the dabbling field forager model, elevating the *dry* threat weight relative to the other threats produced the greatest response for the water quality wetland scenario, improving habitat quality by an additional 8% relative to the base model (Table 3). Increasing the other threat weights decreased the habitat quality score by 2% compared to the base model for the water quality wetland scenario (Table 3). Using different DSWE summarization methods for the *dry* threat and post-model processing resulted in 2% variance in habitat quality scores (Supplemental Table 9).

The bounded effects of drainage modernization and water quality wetland additions for obligate wetland-feeding dabbling ducks in the spring, as discussed above, indicate a maximum habitat quality reduction of 87% and minimum reduction of 54% due to drainage modernization and potential improvements in habitat quality due to water quality wetland additions ranging from four percent to 23% (Figs. 4 and 5).

### Diving Duck Habitat

Diving duck models identified 125 ha of potentially suitable habitat across all modeled watersheds, only seven ha of which occurred within drained depressions. After accounting for threats in the InVEST model, the baseline scenario habitat quality score for diving ducks was 273. Removing depressional areas from the analysis where no DSWE surface water records were identified resulted in a habitat quality score decline of 1%.

Drainage modernization, when all depressions within the modeled watersheds were considered regardless of their hydrologic activity, resulted in a five percent reduction in diving duck habitat quality (Fig. 4; Supplemental Table 9). Reductions in habitat quality occurred as 7 ha of relatively high habitat quality (> 0.15) area shifted to low (≤ 0.01) or zero habitat quality scores after drainage modernization (Supplemental Fig. 4). Reductions in habitat quality when depressional areas with no DSWE identified surface water throughout the study period were removed from the analysis also resulted in a 5% habitat quality reduction (Fig. 5; Supplemental Table 9).

Adding a water quality wetland to all watersheds resulted in a 95% improvement in the habitat quality score across all watersheds when all depressional area was considered in the baseline scenario (Fig. 4). These improvements occurred as 38 ha of modeled area shifted from low (≤ 0.01) or zero habitat quality scores to relatively high (> 0.15) habitat quality scores (Supplemental Fig. 4). Combining drainage modernization with the addition of water quality wetlands and considering all depressional area resulted in a 90% habitat quality score improvement (Fig. 4). When only depressional areas with at least one record of surface water were considered, improvements due to water quality wetlands increased to 96% and 91% when combined with drainage modernization (Fig. 5).

Varying threat weight settings to further bound the effects of landscape management scenarios on diving ducks indicates an additional + 1% and − 1% variability for drainage modernization scenarios and an additional + 188% and − 39% variability for water quality wetland addition scenarios (Table 3). Increasing the *dry* threat weight relative to the other threat weights was responsible for the largest increase in habitat quality in the water quality wetland scenario, while increasing the *wetarea* threat resulted in the largest decline in habitat quality in this scenario (Table 3). Using surface water annual presence/absence or overall frequency for summarizing DSWE surface water extent in model threat layers and post-model processing had no effect on habitat quality scores (Supplemental Table 9).

Accounting for the model variations and sensitivity testing discussed previously, our models bound the effects of drainage modernization on diving ducks and indicate a habitat quality change ranging from − 6% to − 4%, with modeled water quality wetland additions improving habitat quality by 57% to 284% (Figs. 4 and 5).

### Shorebird Habitat

Suitable habitat for shorebirds, based on the baseline land use map and expert opinion, amounted to 1,701 ha, 85% of which occurred within drained depressional areas. According to DSWE analyses of available observations across April and May of the study period, 86% of drained depressional area in the modeled watersheds did not have any records of surface water (Supplemental Fig. 5). After accounting for threats, the baseline habitat quality score in the baseline scenario was 2,838 but declined by 75% to 701 when depressional areas with no records of DSWE surface water were removed from the analysis.

Drainage modernization, like with the dabbling duck models, resulted in an 87% habitat quality reduction when all depressions, regardless of their hydrologic activity, were considered (Fig. 4; Supplemental Table 9). This habitat quality reduction resulted from the shift of 1,439 ha of modeled land from relatively high habitat quality scores (> 0.15) to low (≤ 0.01) or zero values (Supplemental Fig. 4). Considering only watershed depressional area with at least one DSWE observation of surface water resulted in a 47% and 48% reduction in habitat quality, respectively (Fig. 5; Supplemental Table 9).

Water quality wetland additions resulted in only a 1% improvement in habitat quality under the assumption that a one-pixel width (30 m) shoreline would be accessible to these shorebirds in spring (Fig. 4). This resulted from the shift of 60 ha of modeled area from low or zero habitat quality scores to intermediate scores (0.05–0.15) but was accompanied by a net reduction of 44 ha of relatively high quality (> 0.15) habitat (Supplemental Fig. 4). Without available shoreline habitat, water quality wetlands resulted in a decline in habitat quality of 3% due to construction of wetlands in existing shorebird-suitable habitat (Fig. 4). Adding water quality wetlands in combination with drainage improvements, when all depressional areas were included, resulted in an 85–90% habitat quality reduction depending on if shoreline habitat was available (Fig. 4). The habitat quality effect of water quality wetlands relative to the baseline scenario ranged from + 6% to − 11%, depending on if shoreline habitat was available, when depressional areas with no DSWE surface water activity were removed from the analysis (Fig. 5). Combining drainage modernization and water quality wetlands and accounting for depressional areas with no DSWE surface water resulted in a habitat quality reduction between 41 and 58% (Fig. 5).

Varying threat weight settings did not alter the effect of drainage modernization on habitat quality but did increase the benefit of adding water quality wetlands by + 2% relative to the base model when the *dry* threat was elevated (Table 3). Increasing other threat weights had no effect on habitat quality scores compared to the base model. Using surface water annual presence/absence or overall frequency for summarizing DSWE surface water extent in model threat layers and post-model processing had minimal (≤ 1%) effect on habitat quality scores (Supplemental Table 9).

Bounding the potential effects of landscape management scenarios using different threat weight sensitivity tests, DSWE-based removal thresholds, DSWE frequency methods, and water quality wetland shoreline sub-scenarios indicate shorebird habitat quality changes due to drainage modernization ranging from − 90% to − 47% and − 3% to + 8% for water quality wetland scenarios (Figs. 4 and 5).

## Discussion

Our models indicate a potentially large spring migratory benefit of water quality wetland installations for diving ducks, with moderate benefits identified for dabbling ducks and minimal benefit for shorebirds. Combining modeled drainage modernization with water quality wetland additions indicates, with the exception of diving ducks, that habitat quality gains from wetland additions will not come close to offsetting potential habitat quality reductions anticipated in our models due to drainage modernization.

Drained depressional areas that provide surface water in the spring can be highly beneficial for providing food for dabbling ducks and shorebirds during their spring migratory movements (Ballard et al. 2021; Krapu 1974b; Lagrange and Dinsmore 1989; Murphy and Dinsmore 2018b). The value of drained depressional areas for dabbling ducks and shorebirds during spring migration scales to the frequency they pond water because they have limited value when dry. However, since diving ducks require deeper water habitat, the further loss of ponding in these drained shallow depressional areas is unlikely to affect them much. In our analyses we sought to account for surface water frequency in drained depressional areas using DSWE products and penalized areas with less frequent surface water presence while also removing them in certain post-model analyses. Due to limitations with DSWE discussed later, we likely underestimated the extent that some drained wetlands were ponding water. Considering drainage infrastructure bottlenecks are likely to occur at the same time of the year as the spring migration of waterbirds, the desire to improve such infrastructure by farmers indicates that these drained wetlands are periodically ponding water and potentially functioning as existing waterbird habitat.

Modeled drainage modernization dramatically decreased the available suitable habitat for all groups except diving ducks but varied depending on what depressions were considered in the analyses (Figs. 4 and 5). When depressional areas without surface water were considered unsuitable habitat in the baseline scenario, drainage modernization resulted in lower but still large (> 45%) relative reductions in habitat quality for dabbling ducks and shorebirds (Fig. 5).

Modeled water quality wetlands were most beneficial for diving ducks, especially relative to the existing habitat quality in the landscape that offers very few resources for this group, and least beneficial for shorebirds. Like with drainage modernization, however, what depressional areas were considered in the analyses had a large influence on the relative effects of water quality wetland installation (Figs. 4 and 5). This effect resulted from the reduction in suitable habitat in depressional areas in the baseline scenario based on DSWE surface water frequency, which increased the relative gain when water quality wetlands were added to the modeled landscape.

Water quality wetlands as currently designed and installed in the Des Moines Lobe of Iowa do not provide large amounts of shorebird accessible area, typically considered as areas with water depth less than 20 cm. Further, these water quality wetlands are commonly installed in lower-lying areas that may already provide shorebird habitat before the addition of a water quality wetland. The installation of a wetland in these locations may represent a net loss or minimal change in total available shorebird habitat and habitat quality, as evidenced by the observed decrease in habitat quality in water quality scenarios without accessible shorebird shoreline (Figs. 4 and 5). When a one-pixel-width shoreline was in place, these 30 m-wide shorelines represented 56% of the total water quality wetland area across all modeled water quality wetlands, which is likely greater than the typical shallow area installed in Iowa (Iowa Department of Agriculture and Land Stewardship (IDALS), written commun., 2022). The addition of this shoreline accessible area improved habitat quality for spring shorebirds by 1%–8%. Water quality wetlands could be designed and maintained to provide greater benefits for ducks and particularly for shorebirds; for example, if they were not placed in existing shorebird habitat, if they were designed to have a larger shallow water zone around their perimeter, and if they were managed to limit the growth of invasive plants. Of course, design changes might come with tradeoffs such as larger project footprints, a reduction in diving duck habitat, or changes to the wetland’s ability to remove nitrogen. Such potential tradeoffs require additional research to inform decisions focused on maximizing the benefits of water quality wetlands.

As it stands, our models are largely based on expert opinion and interpretation of existing research. As such, further research to validate our models is needed before they are widely applied. This is particularly true when applying these models to other regions, for example the northern Prairie Pothole Region, where existing agricultural drainage is less intense. In these cases, drainage installation or drainage modernization are likely to result in a high degree of consolidation drainage (McCauley et al. 2016, 2015) where smaller, hydrologically dynamic depressional systems are lost in exchange for fewer, more stable lake-like systems that provide much less value to waterbirds (Anteau et al. 2016; Cressey et al. 2016; McKenna et al. 2019). Drainage and water quality wetlands may also have a different effect on waterbirds in other portions of the Prairie Pothole Region or other regions where the landscape is composed of more diverse crops and habitat than the corn and soy dominance that is typical of the southern Prairie Pothole Region. Lastly, our models did not consider other indirect implications of drainage modernization on waterbirds and other organisms, including the potential loss of conservation program lands if drainage is modernized in depressional areas that were converted to conservation program lands primarily due to reduced crop yields.

Our models were also limited by uncertainty in ponding dynamics because they relied heavily on DSWE products for drained depressional hydrology information and assumed that drainage modernization would completely negate the presence of surface water during spring migratory periods. The DSWE products are limited in several ways. First, DSWE is based on 30 m pixel Landsat data products, so wet areas smaller than a pixel or overlapping pixel extents may not be detected (Vanderhoof et al. 2023). Second, the return interval for acquisitions is 16 days, so wetlands with hydroperiods shorter than that could be interpreted as dry. Last, cloudy acquisitions further extend the intervals between acquisitions where a wetland could be ponding water and miss detection. Therefore, the available DSWE products likely underrepresent the degree that wetlands pond water and thus their potential to serve as waterbird habitat during spring migration. If this is indeed the case, then drainage modernization is likely to result in reductions in habitat quality that more closely resemble the reductions when we considered all depressional areas as potential habitat. We did, however, assume the complete loss of surface water in depressional areas after drainage modernization. In reality, the extent and regularity of surface water in drained depressions will depend on local hydrology and the extent of drainage modernization and therefore may not result in the complete loss of surface water.

Our results indicate that drainage modernization will have a large effect on waterbird habitat quality during spring migration unless other beneficial land management practices are simultaneously implemented. The use of controlled drainage, which involves managing the water table height throughout the year to maximize water and nutrient retention and water quality benefits without damaging crop establishment or impeding agricultural practices (Gilliam et al. 1979; Helmers et al. 2022; Skaggs et al. 2012), may provide a rare win–win solution. Controlled drainage may indirectly provide temporary aquatic resources in lower-lying depressional areas for waterbirds in the spring, assuming the water table is set to its highest point during this time (Mitchell et al. 2023), and allow for relatively free-draining soils come time for land preparation and crop establishment. In Iowa, recommended planting dates vary with the weather but have an optimal window for corn from April 11 to May 18 and for soybeans from April 11 to May 20 (Licht and Clemens 2021). Farmers therefore may be able to modernize their drainage systems to effectively remove water from soils during the growing season while retaining water during the non-growing season for waterbird habitat and nutrient retention. Shorebirds, however, may only experience minor habitat benefits from this practice as they are commonly migrating through the region during the optimal planting window in April–May when fields would require free drainage. More research, including stakeholder engagement and field evaluations, could clarify the potential benefits and barriers of using controlled drainage for migratory duck resources.

Drainage modernization aside, combining the results from this study with the findings of others indicates that water quality wetlands are one of the most complete solutions for addressing environmental issues associated with agricultural expansion and intensification (Mitchell et al. 2023). For example, water quality wetlands have the potential to improve water quality (Crumpton et al. 2020), amphibian habitat (Mitchell et al. 2022a), pollinator habitat (Mitchell et al. 2022a), waterbird summer breeding habitat (Otis et al. 2010), and waterbird spring migratory stopover resources. However, many of these studies, including our study, are model-based and thus more field-scale research is required to validate the habitat benefits associated with water quality wetland installations.

## Conclusions

Altogether our models indicate that water quality wetlands are likely to be a beneficial solution for addressing the deterioration and loss of migratory stopover sites for dabbling and diving ducks in the Iowa portion of the Prairie Pothole Region, but they are unlikely to fully offset habitat impairments due to planned drainage modernization projects in the region. However, our models require validation with field observations and should be used with caution before applying to other scenarios and regions. Even so, water quality wetlands represent a relatively comprehensive solution to many of the negative environmental effects associated with agricultural expansion and intensification, and their implementation should be prioritized where appropriate.

## Supplementary Material

Supplementary Material 2

Supplementary Material 1

**Supplementary Information** The online version contains supplementary material available at https://doi.org/10.1007/s13157-025-01930-y.

## Figures and Tables

**Table 1:** Model watershed details for the Iowa portion of the Prairie Pothole Region, USA. WQ = water quality

Catchment ID	County	Watershed Area (ha)	Easement Area (ha)	WQ Wetland Area (ha)	WQ Wetland: Watershed Area (%)	Non-Depressional Corn/Soy (%)	Depressional Corn/Soy (%)	Total Depressional Area (%)	Existing Wetland Area (%)
1	Emmet	322	7	3	0.9	82	9	9	0.6
2	Emmet	287	17	3	0.9	65	19	20	3.6
3	Emmet	250	16	4	1.7	83	3	4	0.9
4	Emmet	498	22	9	1.7	60	0	1	6.7
5	Emmet	184	15	6	3.4	86	3	3	2.1
6	Hancock/Kossuth	1144	16	4	0.4	79	9	9	1.2
7	Kossuth	664	12	8	1.2	76	16	17	2.1
8	Kossuth	732	18	6	0.8	79	14	15	0.5
9	Winnebago	448	11	6	1.3	80	5	5	3.6
10	Winnebago	527	40	13	2.5	72	17	17	2.8
11	Winnebago	242	8	3	1.1	77	2	3	4.2
12	Pocahontas	945	42	7	0.7	84	11	11	0.5
13	Calhoun	651	24	8	1.2	87	8	8	0.5
14	Calhoun	233	9	2	0.9	87	10	10	0.2
15	Humboldt	608	12	3	0.5	81	5	5	2.0
16	Humboldt	432	12	2	0.4	94	2	2	0.8
17	Webster	560	11	2	0.4	83	13	13	0.1
18	Webster	372	11	3	0.7	86	8	8	0.6
19	Wright	560	42	18	3.2	81	6	6	5.0
20	Hamilton	1219	35	15	1.2	75	16	17	0.5
21	Hamilton	388	20	10	2.6	84	3	3	5.8
22	Greene	290	13	4	1.5	92	1	1	0.1
23	Greene	284	15	5	1.6	75	0	0	1.4
24	Greene	281	6	1	0.5	84	8	8	0.4
25	Greene	407	11	3	0.7	90	5	5	0.4
26	Greene/Calhoun	854	18	7	0.8	91	3	3	0.9
27	Boone/Hamilton	1296	26	11	0.9	62	4	4	4.4
28	Boone	328	7	2	0.6	87	8	8	0.5
29	Boone	260	17	4	1.7	93	3	3	0.4
30	Boone	129	25	8	6.4	87	8	8	0.6
31	Story	349	15	4	1.1	91	2	2	0.5
32	Story	531	14	7	1.4	81	10	10	0.7
33	Dallas	274	8	2	0.8	51	1	2	1.7
34	Dallas	593	12	3	0.5	61	5	6	1.2
35	Dallas	508	23	7	1.4	72	8	8	0.4
36	Polk	936	18	7	0.8	85	1	1	0.6
37	Polk	241	9	2	1.0	88	4	4	0.2

**Table 2:** Waterbird group models and representative species in the Iowa portion of the Prairie Pothole Region, USA

Group	Species (Common Name)	Species (*Genus species*)
Dabbling Wetland Obligate Ducks	Gadwall	*Mareca strepera*
	Green-winged teal	*Anas crecca*
	Blue-winged teal	*Spatula discors*
	Northern shoveler	*Spatula clypeata*
	Wood duck	*Aix sponsa*
	Black duck	*Anas rubripes*
Dabbling Field Foraging Ducks	Northern pintail	*Anas acuta*
	American wigeon	*Mareca americana*
	Mallard	*Anas platyrhynchos*
Diving Ducks	Canvasbacks	*Aythya valisineria*
	Lesser scaup	*Aythya affinis*
	Redhead	*Aythya americana*
	Bufflehead	*Bucephala albeola*
	Ring-necked duck	*Aythya collaris*
	Ruddy duck	*Oxyura jamaicensis*
General Shorebird Model	Semipalmated plover	*Charadrius semipalmatus*
	Killdeer	*Charadrius vociferus*
	Hudsonian godwit	*Limosa haemastica*
	Marbled godwit	*Limosa fedoa*
	Stilt sandpiper	*Calidris himantopus*
	Dunlin	*Calidris alpina*
	Baird's sandpiper	*Calidris bairdii*
	Least sandpiper	*Calidris minutilla*
	White-rumped sandpiper	*Calidris fuscicollis*
	Pectoral sandpiper	*Calidris melanotos*
	Semipalmated sandpiper	*Calidris pusilla*
	Spotted sandpiper	*Actitis macularius*
	Solitary sandpiper	*Tringa solitaria*
	Short-billed dowitcher	*Limnodromus griseus*
	Long-billed dowitcher	*Limnodromus scolopaceus*
	Wilson's snipe	*Gallinago delicata*
	Lesser yellowlegs	*Tringa flavipes*
	Greater yellowlegs	*Tringa melanoleuca*
	Willet	*Tringa semipalmata*

**Table 3:** Model threat weight sensitivity test results

Group	Threat Weight Settings	Drainage Modernization	WQ Wetland	Drain. Mod. & WQ Wetland	WQ Wetland, No Shoreline	Drain. Mod. And WQ wetland, No Shoreline
**Dabbling Field Foraging Ducks**	All Threat Weights Equal (DT = 1; CT = 1; RT = 1; RdT = 1; CnT = 1)	−87%	5%	−81%	NA	NA
	High Dry Threat (DT = 3; CT = 0.25; RT = 0.25; RdT = 0.25; CnT = 0.25)	−87%	14%	−72%	NA	NA
	High Crop Threat (DT = 0.25; CT = 3; RT = 0.25; RdT = 0.25; CnT = 0.25)	−87%	4%	−82%	NA	NA
	High Roost Threat (DT = 0.25; CT = 0.25; RT = 3; RdT = 0.25; CnT = 0.25)	−87%	4%	−82%	NA	NA
	High Road Threat (DT = 0.25; CT = 0.25; RT = 0.25; RdT = 3; CnT = 0.25)	−87%	4%	−82%	NA	NA
	High Corn Threat (DT = 0.25; CT = 0.25; RT = 0.25; RdT = 0.25; CnT = 3)	−87%	4%	−82%	NA	NA
**Dabbling Wetland-Foraging Ducks**	All Threat Weights Equal (DT = 1; CT = 1; RT = 1; RdT = 1)	−87%	6%	−81%	NA	NA
	High Dry Threat (DT = 3; CT = 0.33; RT = 0.33; RdT = 0.33)	−87%	14%	−72%	NA	NA
	High Crop Threat (DT = 0.33; CT = 3; RT = 0.33; RdT = 0.33)	−87%	4%	−82%	NA	NA
	High Roost Threat (DT = 0.33; CT = 0.33; RT = 3; RdT = 0.33)	−87%	4%	−82%	NA	NA
	High Road Threat (DT = 0.33; CT = 0.33; RT = 0.33; RdT = 3)	−87%	4%	−82%	NA	NA
**Diving Ducks**	All Threat Weights Equal (DT = 1; CT = 1; RT = 1; RdT = 1; WaT = 1)	−5%	95%	90%	NA	NA
	High Dry Threat (DT = 4; CT = 0.25; RT = 0.25; RdT = 0.25; WaT = 0.25)	−6%	283%	277%	NA	NA
	High Crop Threat (DT = 0.25; CT = 4; RT = 0.25; RdT = 0.25; WaT = 0.25)	−6%	73%	67%	NA	NA
	High Roost Threat (DT = 0.25; CT = 0.25; RT = 4; RdT = 0.25; WaT = 0.25)	−6%	73%	67%	NA	NA
	High Road Threat (DT = 0.25; CT = 0.25; RT = 0.25; RdT = 4; WaT = 0.25)	−6%	74%	68%	NA	NA
	High Area Threat (DT = 0.25; CT = 0.25; RT = 0.25; RdT = 0.25; WaT = 4)	−4%	56%	52%	NA	NA
**Shorebirds**	All Threat Weights Equal (DT = 1; CT = 1; RT = 1; RdT = 1)	−87%	1%	−85%	−3%	−90%
	High Dry Threat (DT = 3; CT = 0.33; RT = 0.33; RdT = 0.33)	−87%	3%	−83%	−3%	−89%
	High Crop Threat (DT = 0.33; CT = 3; RT = 0.33; RdT = 0.33)	−87%	1%	−86%	−3%	−90%
	High Roost Threat (DT = 0.33; CT = 0.33; RT = 3; RdT = 0.33)	−87%	1%	−86%	−3%	−90%
	High Road Threat (DT = 0.33; CT = 0.33; RT = 0.33; RdT = 3)	−87%	1%	−86%	−3%	−90%

DT = Dry threat; CT = Crop threat; RT = Roost threat; RdT = Road threat; CnT = Corn threat; WaT = Wetland area threat; WQ = water quality; NA = not applicable

## Data Availability

Data for constructing and running models are available in the supplemental tables and upon request.
