# Supplementary material for: Modeling Wetland Resources for Spring Migratory Waterbirds Under Different Agricultural Management Scenarios in the Iowa Portion of the Prairie Pothole Region, USA
Source: Wetlands (Wilmington). Author manuscript; Available in PMC 2025 Jun 30. (PMC12207345; doi:10.1007/s13157-025-01930-y)
Supplement: Supplementary Material 2 [file NIHMS2086946-supplement-Supplementary_Material_2.docx]

**Title**: Modeling wetland resources for spring migratory waterbirds under different agricultural management scenarios in the Iowa portion of the Prairie Pothole Region, USA

Target Journal: Wetlands

**Authors:** Mark E. Mitchell^1,2^, Michael J. Anteau^3^, Aaron T. Pearse^3^, Tammy Newcomer-Johnson^4^, Jay Christensen^4^, William Crumpton^5^, Brian Dyson^4^, Timothy J. Canfield^6^, Matthew Helmers^7^, David Green^5^, Kenneth J. Forshay^6^

**Affiliations:
^1^** Oak Ridge Institute for Science and Education (ORISE) Research Participation Program, U.S. Environmental Protection Agency, 26 W. Martin Luther King Drive, Cincinnati, OH 45268, USA

**^2^** Biology Department, Xavier University, 3800 Victory Parkway, Cincinnati, OH 45207, USA; mitchellm36@xavier.edu

**^3^** U.S. Geological Survey, Northern Prairie Wildlife Research Center, 8711 37^th^ Street SE, Jamestown, ND 58401

^4^ Office of Research and Development, U.S. Environmental Protection Agency, 26 W. Martin Luther King Drive, Cincinnati, OH 45268, USA

^5^ Department of Ecology, Evolution, and Organismal Biology, Iowa State University, 251 Bessey Hall, 2200 Osborn Dr., Ames, IA 50011

^6^ Office of Research and Development, U.S. Environmental Protection Agency, 919 Kerr Research Drive, Ada, OK 74820

^7^ Department of Agricultural and Biosystems Engineering, Iowa State University, 4354 Elings, 605 Bissell Rd., Ames, IA 50011

**Supplemental Table 1.** Integrated Valuation of Ecosystem Services and Tradeoffs (InVEST) sensitivity table indicating the relative sensitivity of specific habitat types to specific threats for dabbling field foraging ducks in the Iowa portion of the Prairie Pothole Region, USA. LULC = land use land cover; NLCD = National Land Cover Database.. Unsuitable habitat = 0; potentially suitable habitat = 0.5; maximum potentially suitable habitat = 1.

| LULC | NAME | HABITAT | dry | crop | wetarea | roost | road | corn |
| --- | --- | --- | --- | --- | --- | --- | --- | --- |
| 0 | Background | 0 | 0 | 0 | 0 | 0 | 0 | 0 |
| 1 | Corn | 0 | 0 | 0 | 0 | 0 | 0 | 0 |
| 4 | Sorgum | 0 | 0 | 0 | 0 | 0 | 0 | 0 |
| 5 | Soybeans | 0 | 0 | 0 | 0 | 0 | 0 | 0 |
| 6 | Sunflowers | 0 | 0 | 0 | 0 | 0 | 0 | 0 |
| 12 | Sweet Corn | 0 | 0 | 0 | 0 | 0 | 0 | 0 |
| 13 | Ornamental/Pop Corn | 0 | 0 | 0 | 0 | 0 | 0 | 0 |
| 21 | Barley | 0 | 0 | 0 | 0 | 0 | 0 | 0 |
| 22 | Durum Wheat | 0 | 0 | 0 | 0 | 0 | 0 | 0 |
| 23 | Spring Wheat | 0 | 0 | 0 | 0 | 0 | 0 | 0 |
| 24 | Winter Wheat | 0 | 0 | 0 | 0 | 0 | 0 | 0 |
| 25 | Other Small Grains | 0 | 0 | 0 | 0 | 0 | 0 | 0 |
| 26 | Wheat/Soybeans Doubl Crop | 0 | 0 | 0 | 0 | 0 | 0 | 0 |
| 27 | Rye | 0 | 0 | 0 | 0 | 0 | 0 | 0 |
| 28 | Oats | 0 | 0 | 0 | 0 | 0 | 0 | 0 |
| 29 | Millet | 0 | 0 | 0 | 0 | 0 | 0 | 0 |
| 31 | Canola | 0 | 0 | 0 | 0 | 0 | 0 | 0 |
| 32 | Flaxseed | 0 | 0 | 0 | 0 | 0 | 0 | 0 |
| 33 | Safflower | 0 | 0 | 0 | 0 | 0 | 0 | 0 |
| 34 | Rape Seed | 0 | 0 | 0 | 0 | 0 | 0 | 0 |
| 35 | Mustard | 0 | 0 | 0 | 0 | 0 | 0 | 0 |
| 36 | Alfalfa | 0 | 0 | 0 | 0 | 0 | 0 | 0 |
| 37 | Other Hays | 0 | 0 | 0 | 0 | 0 | 0 | 0 |
| 38 | Camelina | 0 | 0 | 0 | 0 | 0 | 0 | 0 |
| 39 | Seed Crop | 0 | 0 | 0 | 0 | 0 | 0 | 0 |
| 41 | Sugarbeets | 0 | 0 | 0 | 0 | 0 | 0 | 0 |
| 42 | Dry Beans | 0 | 0 | 0 | 0 | 0 | 0 | 0 |
| 43 | Potatoes | 0 | 0 | 0 | 0 | 0 | 0 | 0 |
| 44 | Other Crops | 0 | 0 | 0 | 0 | 0 | 0 | 0 |
| 47 | Misc Veg/Fruits | 0 | 0 | 0 | 0 | 0 | 0 | 0 |
| 52 | Lentils | 0 | 0 | 0 | 0 | 0 | 0 | 0 |
| 53 | Peas | 0 | 0 | 0 | 0 | 0 | 0 | 0 |
| 57 | Herbs | 0 | 0 | 0 | 0 | 0 | 0 | 0 |
| 58 | Clover/Wildflowers | 0 | 0 | 0 | 0 | 0 | 0 | 0 |
| 59 | Seed/Sod Grass | 0 | 0 | 0 | 0 | 0 | 0 | 0 |
| 60 | Switchgrass | 0 | 0 | 0 | 0 | 0 | 0 | 0 |
| 61 | Fallow/Idle Cropland | 0 | 0 | 0 | 0 | 0 | 0 | 0 |
| 62 | Pasture/Range/CRP/Non-Ag | 0 | 0 | 0 | 0 | 0 | 0 | 0 |
| 63 | Woodland | 0 | 0 | 0 | 0 | 0 | 0 | 0 |
| 64 | Shrubland | 0 | 0 | 0 | 0 | 0 | 0 | 0 |
| 65 | Barren | 0 | 0 | 0 | 0 | 0 | 0 | 0 |
| 68 | Apples | 0 | 0 | 0 | 0 | 0 | 0 | 0 |
| 69 | Grapes | 0 | 0 | 0 | 0 | 0 | 0 | 0 |
| 70 | Christmas Trees | 0 | 0 | 0 | 0 | 0 | 0 | 0 |
| 82 | Urban/Developed | 0 | 0 | 0 | 0 | 0 | 0 | 0 |
| 83 | Water | 1 | 1 | 1 | 0 | 1 | 1 | 1 |
| 87 | Wetlands | 1 | 1 | 1 | 0 | 1 | 1 | 1 |
| 88 | Non-ag/Undefined | 0 | 0 | 0 | 0 | 0 | 0 | 0 |
| 111 | NLCD-Open Water | 0 | 0 | 0 | 0 | 0 | 0 | 0 |
| 121 | NLCD-Open Space | 0 | 0 | 0 | 0 | 0 | 0 | 0 |
| 122 | NLCD-Developed/Low Intensity | 0 | 0 | 0 | 0 | 0 | 0 | 0 |
| 123 | NLCD-Developed/Medium Intensity | 0 | 0 | 0 | 0 | 0 | 0 | 0 |
| 124 | NLCD-Developed/High Intensity | 0 | 0 | 0 | 0 | 0 | 0 | 0 |
| 131 | NLCD-Barren | 0 | 0 | 0 | 0 | 0 | 0 | 0 |
| 141 | NLCD-Deciduous Forest | 0 | 0 | 0 | 0 | 0 | 0 | 0 |
| 142 | NLCD-Evergreen Forest | 0 | 0 | 0 | 0 | 0 | 0 | 0 |
| 143 | NLCD-Mixed Forest | 0 | 0 | 0 | 0 | 0 | 0 | 0 |
| 152 | NLCD-Shrubland | 0 | 0 | 0 | 0 | 0 | 0 | 0 |
| 171 | NLCD-Grassland Herbaceous | 0 | 0 | 0 | 0 | 0 | 0 | 0 |
| 176 | Grassland/Pasture | 0 | 0 | 0 | 0 | 0 | 0 | 0 |
| 181 | NLCD-Pasture Hayland | 0 | 0 | 0 | 0 | 0 | 0 | 0 |
| 182 | NLCD-Croplands | 0 | 0 | 0 | 0 | 0 | 0 | 0 |
| 190 | NLCD-Woody Wetland | 0.5 | 1 | 1 | 0 | 1 | 1 | 1 |
| 195 | NLCD-Herbaceous Wetland | 1 | 1 | 1 | 0 | 1 | 1 | 1 |
| 205 | Triticale | 0 | 0 | 0 | 0 | 0 | 0 | 0 |
| 221 | Strawberries | 0 | 0 | 0 | 0 | 0 | 0 | 0 |
| 224 | Vetch | 0 | 0 | 0 | 0 | 0 | 0 | 0 |
| 225 | Dbl. Crop WinWht/Corn | 0 | 0 | 0 | 0 | 0 | 0 | 0 |
| 229 | Pumpkins | 0 | 0 | 0 | 0 | 0 | 0 | 0 |
| 236 | Dbl. Crop WinWht/Sorghum | 0 | 0 | 0 | 0 | 0 | 0 | 0 |
| 240 | Dbl. Crop Soybeans/Oats | 0 | 0 | 0 | 0 | 0 | 0 | 0 |
| 241 | Dbl. Crop Corn/Soybeans | 0 | 0 | 0 | 0 | 0 | 0 | 0 |
| 246 | Radishes | 0 | 0 | 0 | 0 | 0 | 0 | 0 |
| 401 | Temporary Wetland | 1 | 1 | 1 | 0 | 1 | 1 | 1 |
| 402 | Seasonal Wetland | 1 | 1 | 1 | 0 | 1 | 1 | 1 |
| 403 | Semipermanent Wetland | 0.5 | 1 | 1 | 0 | 1 | 1 | 1 |
| 404 | Permanent Wetlands | 0.5 | 1 | 1 | 0 | 1 | 1 | 1 |
| 405 | Lacustrine | 0 | 0 | 0 | 0 | 0 | 0 | 0 |
| 406 | Riverine Non Intermittent | 0 | 0 | 0 | 0 | 0 | 0 | 0 |
| 407 | Artificially Flooded Wetland | 0.5 | 1 | 1 | 0 | 1 | 1 | 1 |
| 408 | Wetland 160 M Buffer | 0 | 0 | 0 | 0 | 0 | 0 | 0 |
| 411 | WQ wetland | 0.5 | 1 | 1 | 0 | 1 | 1 | 1 |
| 412 | WQ easement | 0 | 0 | 0 | 0 | 0 | 0 | 0 |
| 413 | WQ Wetland 160m buffer habitat | 0 | 0 | 0 | 0 | 0 | 0 | 0 |
| 600 | Grassland Wetland | 0 | 0 | 0 | 0 | 0 | 0 | 0 |
| 601 | Road | 0 | 0 | 0 | 0 | 0 | 0 | 0 |
| 602 | Lacustrine Perimeter | 0.5 | 1 | 1 | 0 | 1 | 1 | 1 |
| 603 | Riverine Intermittent Streams | 0.5 | 1 | 1 | 0 | 1 | 1 | 1 |
| 604 | WQ Wetland Perimeter | 0.5 | 1 | 1 | 0 | 1 | 1 | 1 |
| 1001 | Depression Corn | 1 | 1 | 1 | 0 | 1 | 1 | 1 |
| 1005 | Depression Soy | 1 | 1 | 1 | 0 | 1 | 1 | 1 |
| 1012 | Depression Sweet Corn | 1 | 1 | 1 | 0 | 1 | 1 | 1 |
| 1013 | Depression Ornamental/Pop Corn | 1 | 1 | 1 | 0 | 1 | 1 | 1 |
| 1022 | Depression Durum Wheat | 1 | 1 | 1 | 0 | 1 | 1 | 1 |
| 1024 | Depression Winter Wheat | 1 | 1 | 1 | 0 | 1 | 1 | 1 |
| 1027 | Depression Rye | 1 | 1 | 1 | 0 | 1 | 1 | 1 |
| 1028 | Depression Oats | 1 | 1 | 1 | 0 | 1 | 1 | 1 |
| 1036 | Depression Alfalfa | 1 | 1 | 1 | 0 | 1 | 1 | 1 |
| 1037 | Depression Other Hays | 1 | 1 | 1 | 0 | 1 | 1 | 1 |
| 1059 | Depression Seed/Sod Grass | 1 | 1 | 1 | 0 | 1 | 1 | 1 |
| 1061 | Depression Fallow/Idle Cropland | 1 | 1 | 1 | 0 | 1 | 1 | 1 |
| 1111 | Depression NLCD-Open Water | 1 | 1 | 1 | 0 | 1 | 1 | 1 |
| 1121 | Depression NLCD-Open Space | 1 | 1 | 1 | 0 | 1 | 1 | 1 |
| 1122 | Depression NLCD-Developed/Low Intensity | 0 | 0 | 0 | 0 | 0 | 0 | 0 |
| 1123 | Depression NLCD-Developed/Medium Intensity | 0 | 0 | 0 | 0 | 0 | 0 | 0 |
| 1124 | Depression NLCD-Developed/High Intensity | 0 | 0 | 0 | 0 | 0 | 0 | 0 |
| 1131 | Depression NLCD-Barren | 1 | 1 | 1 | 0 | 1 | 1 | 1 |
| 1141 | Depression NLCD-Deciduous Forest | 1 | 1 | 1 | 0 | 1 | 1 | 1 |
| 1142 | Depression NLCD-Evergreen Forest | 1 | 1 | 1 | 0 | 1 | 1 | 1 |
| 1143 | Depression NLCD-Mixed Forest | 1 | 1 | 1 | 0 | 1 | 1 | 1 |
| 1152 | Depression NLCD-Shrubland | 1 | 1 | 1 | 0 | 1 | 1 | 1 |
| 1176 | Depression Grassland/Pasture | 1 | 1 | 1 | 0 | 1 | 1 | 1 |
| 1190 | Depression NLCD-Woody Wetland | 1 | 1 | 1 | 0 | 1 | 1 | 1 |
| 1195 | Depression NLCD-Herbaceous Wetland | 1 | 1 | 1 | 0 | 1 | 1 | 1 |

**Supplemental Table 2.** Integrated Valuation of Ecosystem Services and Tradeoffs (InVEST) threats table for dabbling field foraging ducks in the Iowa portion of the Prairie Pothole Region, USA.

| THREAT | MAX_DIST | WEIGHT | DECAY | BASE_PATH | CUR_PATH | FUT_PATH |
| --- | --- | --- | --- | --- | --- | --- |
| dry | 0.1 | 1 | linear |  | dry_c.tif |  |
| crop | 1 | 1 | linear |  | crop_c.tif |  |
| roost | 18.5 | 1 | linear |  | roost_c.tif |  |
| road | 0.5 | 1 | linear |  | road_c.tif |  |
| corn | 5 | 1 | linear |  | corn_c.tif |  |

**Supplemental Table 3.** Integrated Valuation of Ecosystem Services and Tradeoffs (InVEST) sensitivity table indicating the relative sensitivity of specific habitat types to specific threats for for dabbling obligate wetland foraging ducks in the Iowa portion of the Prairie Pothole Region, USA. LULC = land use land cover; NLCD = National land cover database. Unsuitable habitat = 0; potentially suitable habitat = 0.5; maximum potentially suitable habitat = 1.

| LULC | NAME | HABITAT | dry | crop | wetarea | roost | road | corn |
| --- | --- | --- | --- | --- | --- | --- | --- | --- |
| 0 | Background | 0 | 0 | 0 | 0 | 0 | 0 | 0 |
| 1 | Corn | 0 | 0 | 0 | 0 | 0 | 0 | 0 |
| 4 | Sorgum | 0 | 0 | 0 | 0 | 0 | 0 | 0 |
| 5 | Soybeans | 0 | 0 | 0 | 0 | 0 | 0 | 0 |
| 6 | Sunflowers | 0 | 0 | 0 | 0 | 0 | 0 | 0 |
| 12 | Sweet Corn | 0 | 0 | 0 | 0 | 0 | 0 | 0 |
| 13 | Ornamental/Pop Corn | 0 | 0 | 0 | 0 | 0 | 0 | 0 |
| 21 | Barley | 0 | 0 | 0 | 0 | 0 | 0 | 0 |
| 22 | Durum Wheat | 0 | 0 | 0 | 0 | 0 | 0 | 0 |
| 23 | Spring Wheat | 0 | 0 | 0 | 0 | 0 | 0 | 0 |
| 24 | Winter Wheat | 0 | 0 | 0 | 0 | 0 | 0 | 0 |
| 25 | Other Small Grains | 0 | 0 | 0 | 0 | 0 | 0 | 0 |
| 26 | Wheat/Soybeans Doubl Crop | 0 | 0 | 0 | 0 | 0 | 0 | 0 |
| 27 | Rye | 0 | 0 | 0 | 0 | 0 | 0 | 0 |
| 28 | Oats | 0 | 0 | 0 | 0 | 0 | 0 | 0 |
| 29 | Millet | 0 | 0 | 0 | 0 | 0 | 0 | 0 |
| 31 | Canola | 0 | 0 | 0 | 0 | 0 | 0 | 0 |
| 32 | Flaxseed | 0 | 0 | 0 | 0 | 0 | 0 | 0 |
| 33 | Safflower | 0 | 0 | 0 | 0 | 0 | 0 | 0 |
| 34 | Rape Seed | 0 | 0 | 0 | 0 | 0 | 0 | 0 |
| 35 | Mustard | 0 | 0 | 0 | 0 | 0 | 0 | 0 |
| 36 | Alfalfa | 0 | 0 | 0 | 0 | 0 | 0 | 0 |
| 37 | Other Hays | 0 | 0 | 0 | 0 | 0 | 0 | 0 |
| 38 | Camelina | 0 | 0 | 0 | 0 | 0 | 0 | 0 |
| 39 | Seed Crop | 0 | 0 | 0 | 0 | 0 | 0 | 0 |
| 41 | Sugarbeets | 0 | 0 | 0 | 0 | 0 | 0 | 0 |
| 42 | Dry Beans | 0 | 0 | 0 | 0 | 0 | 0 | 0 |
| 43 | Potatoes | 0 | 0 | 0 | 0 | 0 | 0 | 0 |
| 44 | Other Crops | 0 | 0 | 0 | 0 | 0 | 0 | 0 |
| 47 | Misc Veg/Fruits | 0 | 0 | 0 | 0 | 0 | 0 | 0 |
| 52 | Lentils | 0 | 0 | 0 | 0 | 0 | 0 | 0 |
| 53 | Peas | 0 | 0 | 0 | 0 | 0 | 0 | 0 |
| 57 | Herbs | 0 | 0 | 0 | 0 | 0 | 0 | 0 |
| 58 | Clover/Wildflowers | 0 | 0 | 0 | 0 | 0 | 0 | 0 |
| 59 | Seed/Sod Grass | 0 | 0 | 0 | 0 | 0 | 0 | 0 |
| 60 | Switchgrass | 0 | 0 | 0 | 0 | 0 | 0 | 0 |
| 61 | Fallow/Idle Cropland | 0 | 0 | 0 | 0 | 0 | 0 | 0 |
| 62 | Pasture/Range/CRP/Non-Ag | 0 | 0 | 0 | 0 | 0 | 0 | 0 |
| 63 | Woodland | 0 | 0 | 0 | 0 | 0 | 0 | 0 |
| 64 | Shrubland | 0 | 0 | 0 | 0 | 0 | 0 | 0 |
| 65 | Barren | 0 | 0 | 0 | 0 | 0 | 0 | 0 |
| 68 | Apples | 0 | 0 | 0 | 0 | 0 | 0 | 0 |
| 69 | Grapes | 0 | 0 | 0 | 0 | 0 | 0 | 0 |
| 70 | Christmas Trees | 0 | 0 | 0 | 0 | 0 | 0 | 0 |
| 82 | Urban/Developed | 0 | 0 | 0 | 0 | 0 | 0 | 0 |
| 83 | Water | 1 | 1 | 1 | 0 | 1 | 1 | 0 |
| 87 | Wetlands | 1 | 1 | 1 | 0 | 1 | 1 | 0 |
| 88 | Non-ag/Undefined | 0 | 0 | 0 | 0 | 0 | 0 | 0 |
| 111 | NLCD-Open Water | 0 | 0 | 0 | 0 | 0 | 0 | 0 |
| 121 | NLCD-Open Space | 0 | 0 | 0 | 0 | 0 | 0 | 0 |
| 122 | NLCD-Developed/Low Intensity | 0 | 0 | 0 | 0 | 0 | 0 | 0 |
| 123 | NLCD-Developed/Medium Intensity | 0 | 0 | 0 | 0 | 0 | 0 | 0 |
| 124 | NLCD-Developed/High Intensity | 0 | 0 | 0 | 0 | 0 | 0 | 0 |
| 131 | NLCD-Barren | 0 | 0 | 0 | 0 | 0 | 0 | 0 |
| 141 | NLCD-Deciduous Forest | 0 | 0 | 0 | 0 | 0 | 0 | 0 |
| 142 | NLCD-Evergreen Forest | 0 | 0 | 0 | 0 | 0 | 0 | 0 |
| 143 | NLCD-Mixed Forest | 0 | 0 | 0 | 0 | 0 | 0 | 0 |
| 152 | NLCD-Shrubland | 0 | 0 | 0 | 0 | 0 | 0 | 0 |
| 171 | NLCD-Grassland Herbaceous | 0 | 0 | 0 | 0 | 0 | 0 | 0 |
| 176 | Grassland/Pasture | 0 | 0 | 0 | 0 | 0 | 0 | 0 |
| 181 | NLCD-Pasture Hayland | 0 | 0 | 0 | 0 | 0 | 0 | 0 |
| 182 | NLCD-Croplands | 0 | 0 | 0 | 0 | 0 | 0 | 0 |
| 190 | NLCD-Woody Wetland | 0.5 | 1 | 1 | 0 | 1 | 1 | 0 |
| 195 | NLCD-Herbaceous Wetland | 1 | 1 | 1 | 0 | 1 | 1 | 0 |
| 205 | Triticale | 0 | 0 | 0 | 0 | 0 | 0 | 0 |
| 221 | Strawberries | 0 | 0 | 0 | 0 | 0 | 0 | 0 |
| 224 | Vetch | 0 | 0 | 0 | 0 | 0 | 0 | 0 |
| 225 | Dbl. Crop WinWht/Corn | 0 | 0 | 0 | 0 | 0 | 0 | 0 |
| 229 | Pumpkins | 0 | 0 | 0 | 0 | 0 | 0 | 0 |
| 236 | Dbl. Crop WinWht/Sorghum | 0 | 0 | 0 | 0 | 0 | 0 | 0 |
| 240 | Dbl. Crop Soybeans/Oats | 0 | 0 | 0 | 0 | 0 | 0 | 0 |
| 241 | Dbl. Crop Corn/Soybeans | 0 | 0 | 0 | 0 | 0 | 0 | 0 |
| 246 | Radishes | 0 | 0 | 0 | 0 | 0 | 0 | 0 |
| 401 | Temporary Wetland | 1 | 1 | 1 | 0 | 1 | 1 | 0 |
| 402 | Seasonal Wetland | 1 | 1 | 1 | 0 | 1 | 1 | 0 |
| 403 | Semipermanent Wetland | 0.5 | 1 | 1 | 0 | 1 | 1 | 0 |
| 404 | Permanent Wetlands | 0.5 | 1 | 1 | 0 | 1 | 1 | 0 |
| 405 | Lacustrine | 0 | 0 | 0 | 0 | 0 | 0 | 0 |
| 406 | Riverine Non Intermittent | 0 | 0 | 0 | 0 | 0 | 0 | 0 |
| 407 | Artificially Flooded Wetland | 0.5 | 1 | 1 | 0 | 1 | 1 | 0 |
| 408 | Wetland 160 M Buffer | 0 | 0 | 0 | 0 | 0 | 0 | 0 |
| 411 | WQ wetland | 0.5 | 1 | 1 | 0 | 1 | 1 | 0 |
| 412 | WQ easement | 0 | 0 | 0 | 0 | 0 | 0 | 0 |
| 413 | WQ Wetland 160m buffer habitat | 0 | 0 | 0 | 0 | 0 | 0 | 0 |
| 600 | Grassland Wetland | 0 | 0 | 0 | 0 | 0 | 0 | 0 |
| 601 | Road | 0 | 0 | 0 | 0 | 0 | 0 | 0 |
| 602 | Lacustrine Perimeter | 0.5 | 1 | 1 | 0 | 1 | 1 | 0 |
| 603 | Riverine Intermittent Streams | 0.5 | 1 | 1 | 0 | 1 | 1 | 0 |
| 604 | WQ Wetland Perimeter | 0.5 | 1 | 1 | 0 | 1 | 1 | 0 |
| 1001 | Depression Corn | 1 | 1 | 1 | 0 | 1 | 1 | 0 |
| 1005 | Depression Soy | 1 | 1 | 1 | 0 | 1 | 1 | 0 |
| 1012 | Depression Sweet Corn | 1 | 1 | 1 | 0 | 1 | 1 | 0 |
| 1013 | Depression Ornamental/Pop Corn | 1 | 1 | 1 | 0 | 1 | 1 | 0 |
| 1022 | Depression Durum Wheat | 1 | 1 | 1 | 0 | 1 | 1 | 0 |
| 1024 | Depression Winter Wheat | 1 | 1 | 1 | 0 | 1 | 1 | 0 |
| 1027 | Depression Rye | 1 | 1 | 1 | 0 | 1 | 1 | 0 |
| 1028 | Depression Oats | 1 | 1 | 1 | 0 | 1 | 1 | 0 |
| 1036 | Depression Alfalfa | 1 | 1 | 1 | 0 | 1 | 1 | 0 |
| 1037 | Depression Other Hays | 1 | 1 | 1 | 0 | 1 | 1 | 0 |
| 1059 | Depression Seed/Sod Grass | 1 | 1 | 1 | 0 | 1 | 1 | 0 |
| 1061 | Depression Fallow/Idle Cropland | 1 | 1 | 1 | 0 | 1 | 1 | 0 |
| 1111 | Depression NLCD-Open Water | 1 | 1 | 1 | 0 | 1 | 1 | 0 |
| 1121 | Depression NLCD-Open Space | 1 | 1 | 1 | 0 | 1 | 1 | 0 |
| 1122 | Depression NLCD-Developed/Low Intensity | 0 | 0 | 0 | 0 | 0 | 0 | 0 |
| 1123 | Depression NLCD-Developed/Medium Intensity | 0 | 0 | 0 | 0 | 0 | 0 | 0 |
| 1124 | Depression NLCD-Developed/High Intensity | 0 | 0 | 0 | 0 | 0 | 0 | 0 |
| 1131 | Depression NLCD-Barren | 1 | 1 | 1 | 0 | 1 | 1 | 0 |
| 1141 | Depression NLCD-Deciduous Forest | 1 | 1 | 1 | 0 | 1 | 1 | 0 |
| 1142 | Depression NLCD-Evergreen Forest | 1 | 1 | 1 | 0 | 1 | 1 | 0 |
| 1143 | Depression NLCD-Mixed Forest | 1 | 1 | 1 | 0 | 1 | 1 | 0 |
| 1152 | Depression NLCD-Shrubland | 1 | 1 | 1 | 0 | 1 | 1 | 0 |
| 1176 | Depression Grassland/Pasture | 1 | 1 | 1 | 0 | 1 | 1 | 0 |
| 1190 | Depression NLCD-Woody Wetland | 1 | 1 | 1 | 0 | 1 | 1 | 0 |
| 1195 | Depression NLCD-Herbaceous Wetland | 1 | 1 | 1 | 0 | 1 | 1 | 0 |

**Supplemental Table 4.** Integrated Valuation of Ecosystem Services and Tradeoffs (InVEST) threats table for dabbling obligate wetland foraging ducks in the Iowa portion of the Prairie Pothole Region, USA.

| THREAT | MAX_DIST | WEIGHT | DECAY | BASE_PATH | CUR_PATH | FUT_PATH |
| --- | --- | --- | --- | --- | --- | --- |
| dry | 0.1 | 1 | linear |  | dry_c.tif |  |
| crop | 1 | 1 | linear |  | crop_c.tif |  |
| roost | 5 | 1 | linear |  | roost_c.tif |  |
| road | 0.5 | 1 | linear |  | road_c.tif |  |

**Supplemental Table 5.** Integrated Valuation of Ecosystem Services and Tradeoffs (InVEST) sensitivity table indicating the relative sensitivity of specific habitat types to specific threats for diving ducks in the Iowa portion of the Prairie Pothole Region, USA. LULC = land use land cover; NLCD = National land cover database. Unsuitable habitat = 0; potentially suitable habitat = 0.5; maximum potentially suitable habitat = 1.

| LULC | NAME | HABITAT | dry | crop | wetarea | roost | road | corn |
| --- | --- | --- | --- | --- | --- | --- | --- | --- |
| 0 | Background | 0 | 0 | 0 | 0 | 0 | 0 | 0 |
| 1 | Corn | 0 | 0 | 0 | 0 | 0 | 0 | 0 |
| 4 | Sorgum | 0 | 0 | 0 | 0 | 0 | 0 | 0 |
| 5 | Soybeans | 0 | 0 | 0 | 0 | 0 | 0 | 0 |
| 6 | Sunflowers | 0 | 0 | 0 | 0 | 0 | 0 | 0 |
| 12 | Sweet Corn | 0 | 0 | 0 | 0 | 0 | 0 | 0 |
| 13 | Ornamental/Pop Corn | 0 | 0 | 0 | 0 | 0 | 0 | 0 |
| 21 | Barley | 0 | 0 | 0 | 0 | 0 | 0 | 0 |
| 22 | Durum Wheat | 0 | 0 | 0 | 0 | 0 | 0 | 0 |
| 23 | Spring Wheat | 0 | 0 | 0 | 0 | 0 | 0 | 0 |
| 24 | Winter Wheat | 0 | 0 | 0 | 0 | 0 | 0 | 0 |
| 25 | Other Small Grains | 0 | 0 | 0 | 0 | 0 | 0 | 0 |
| 26 | Wheat/Soybeans Doubl Crop | 0 | 0 | 0 | 0 | 0 | 0 | 0 |
| 27 | Rye | 0 | 0 | 0 | 0 | 0 | 0 | 0 |
| 28 | Oats | 0 | 0 | 0 | 0 | 0 | 0 | 0 |
| 29 | Millet | 0 | 0 | 0 | 0 | 0 | 0 | 0 |
| 31 | Canola | 0 | 0 | 0 | 0 | 0 | 0 | 0 |
| 32 | Flaxseed | 0 | 0 | 0 | 0 | 0 | 0 | 0 |
| 33 | Safflower | 0 | 0 | 0 | 0 | 0 | 0 | 0 |
| 34 | Rape Seed | 0 | 0 | 0 | 0 | 0 | 0 | 0 |
| 35 | Mustard | 0 | 0 | 0 | 0 | 0 | 0 | 0 |
| 36 | Alfalfa | 0 | 0 | 0 | 0 | 0 | 0 | 0 |
| 37 | Other Hays | 0 | 0 | 0 | 0 | 0 | 0 | 0 |
| 38 | Camelina | 0 | 0 | 0 | 0 | 0 | 0 | 0 |
| 39 | Seed Crop | 0 | 0 | 0 | 0 | 0 | 0 | 0 |
| 41 | Sugarbeets | 0 | 0 | 0 | 0 | 0 | 0 | 0 |
| 42 | Dry Beans | 0 | 0 | 0 | 0 | 0 | 0 | 0 |
| 43 | Potatoes | 0 | 0 | 0 | 0 | 0 | 0 | 0 |
| 44 | Other Crops | 0 | 0 | 0 | 0 | 0 | 0 | 0 |
| 47 | Misc Veg/Fruits | 0 | 0 | 0 | 0 | 0 | 0 | 0 |
| 52 | Lentils | 0 | 0 | 0 | 0 | 0 | 0 | 0 |
| 53 | Peas | 0 | 0 | 0 | 0 | 0 | 0 | 0 |
| 57 | Herbs | 0 | 0 | 0 | 0 | 0 | 0 | 0 |
| 58 | Clover/Wildflowers | 0 | 0 | 0 | 0 | 0 | 0 | 0 |
| 59 | Seed/Sod Grass | 0 | 0 | 0 | 0 | 0 | 0 | 0 |
| 60 | Switchgrass | 0 | 0 | 0 | 0 | 0 | 0 | 0 |
| 61 | Fallow/Idle Cropland | 0 | 0 | 0 | 0 | 0 | 0 | 0 |
| 62 | Pasture/Range/CRP/Non-Ag | 0 | 0 | 0 | 0 | 0 | 0 | 0 |
| 63 | Woodland | 0 | 0 | 0 | 0 | 0 | 0 | 0 |
| 64 | Shrubland | 0 | 0 | 0 | 0 | 0 | 0 | 0 |
| 65 | Barren | 0 | 0 | 0 | 0 | 0 | 0 | 0 |
| 68 | Apples | 0 | 0 | 0 | 0 | 0 | 0 | 0 |
| 69 | Grapes | 0 | 0 | 0 | 0 | 0 | 0 | 0 |
| 70 | Christmas Trees | 0 | 0 | 0 | 0 | 0 | 0 | 0 |
| 82 | Urban/Developed | 0 | 0 | 0 | 0 | 0 | 0 | 0 |
| 83 | Water | 0 | 0 | 0 | 0 | 0 | 0 | 0 |
| 87 | Wetlands | 0 | 0 | 0 | 0 | 0 | 0 | 0 |
| 88 | Non-ag/Undefined | 0 | 0 | 0 | 0 | 0 | 0 | 0 |
| 111 | NLCD-Open Water | 0 | 0 | 0 | 0 | 0 | 0 | 0 |
| 121 | NLCD-Open Space | 0 | 0 | 0 | 0 | 0 | 0 | 0 |
| 122 | NLCD-Developed/Low Intensity | 0 | 0 | 0 | 0 | 0 | 0 | 0 |
| 123 | NLCD-Developed/Medium Intensity | 0 | 0 | 0 | 0 | 0 | 0 | 0 |
| 124 | NLCD-Developed/High Intensity | 0 | 0 | 0 | 0 | 0 | 0 | 0 |
| 131 | NLCD-Barren | 0 | 0 | 0 | 0 | 0 | 0 | 0 |
| 141 | NLCD-Deciduous Forest | 0 | 0 | 0 | 0 | 0 | 0 | 0 |
| 142 | NLCD-Evergreen Forest | 0 | 0 | 0 | 0 | 0 | 0 | 0 |
| 143 | NLCD-Mixed Forest | 0 | 0 | 0 | 0 | 0 | 0 | 0 |
| 152 | NLCD-Shrubland | 0 | 0 | 0 | 0 | 0 | 0 | 0 |
| 171 | NLCD-Grassland Herbaceous | 0 | 0 | 0 | 0 | 0 | 0 | 0 |
| 176 | Grassland/Pasture | 0 | 0 | 0 | 0 | 0 | 0 | 0 |
| 181 | NLCD-Pasture Hayland | 0 | 0 | 0 | 0 | 0 | 0 | 0 |
| 182 | NLCD-Croplands | 0 | 0 | 0 | 0 | 0 | 0 | 0 |
| 190 | NLCD-Woody Wetland | 0 | 0 | 0 | 0 | 0 | 0 | 0 |
| 195 | NLCD-Herbaceous Wetland | 0 | 0 | 0 | 0 | 0 | 0 | 0 |
| 205 | Triticale | 0 | 0 | 0 | 0 | 0 | 0 | 0 |
| 221 | Strawberries | 0 | 0 | 0 | 0 | 0 | 0 | 0 |
| 224 | Vetch | 0 | 0 | 0 | 0 | 0 | 0 | 0 |
| 225 | Dbl. Crop WinWht/Corn | 0 | 0 | 0 | 0 | 0 | 0 | 0 |
| 229 | Pumpkins | 0 | 0 | 0 | 0 | 0 | 0 | 0 |
| 236 | Dbl. Crop WinWht/Sorghum | 0 | 0 | 0 | 0 | 0 | 0 | 0 |
| 240 | Dbl. Crop Soybeans/Oats | 0 | 0 | 0 | 0 | 0 | 0 | 0 |
| 241 | Dbl. Crop Corn/Soybeans | 0 | 0 | 0 | 0 | 0 | 0 | 0 |
| 246 | Radishes | 0 | 0 | 0 | 0 | 0 | 0 | 0 |
| 401 | Temporary Wetland | 0 | 0 | 0 | 0 | 0 | 0 | 0 |
| 402 | Seasonal Wetland | 1 | 1 | 1 | 1 | 1 | 1 | 0 |
| 403 | Semipermanent Wetland | 1 | 1 | 1 | 1 | 1 | 1 | 0 |
| 404 | Permanent Wetlands | 1 | 1 | 1 | 1 | 1 | 1 | 0 |
| 405 | Lacustrine | 0.5 | 1 | 1 | 1 | 1 | 1 | 0 |
| 406 | Riverine Non Intermittent | 0.5 | 1 | 1 | 1 | 1 | 1 | 0 |
| 407 | Artificially Flooded Wetland | 1 | 1 | 1 | 1 | 1 | 1 | 0 |
| 408 | Wetland 160 M Buffer | 0 | 0 | 0 | 0 | 0 | 0 | 0 |
| 411 | WQ wetland | 1 | 1 | 1 | 1 | 1 | 1 | 0 |
| 412 | WQ easement | 0 | 0 | 0 | 0 | 0 | 0 | 0 |
| 413 | WQ Wetland 160m buffer habitat | 0 | 0 | 0 | 0 | 0 | 0 | 0 |
| 600 | Grassland Wetland | 0 | 0 | 0 | 0 | 0 | 0 | 0 |
| 601 | Road | 0 | 0 | 0 | 0 | 0 | 0 | 0 |
| 602 | Lacustrine Perimeter | 0 | 0 | 0 | 0 | 0 | 0 | 0 |
| 603 | Riverine Intermittent Streams | 0 | 0 | 0 | 0 | 0 | 0 | 0 |
| 604 | WQ Wetland Perimeter | 0 | 0 | 0 | 0 | 0 | 0 | 0 |
| 1001 | Depression Corn | 0 | 0 | 0 | 0 | 0 | 0 | 0 |
| 1005 | Depression Soy | 0 | 0 | 0 | 0 | 0 | 0 | 0 |
| 1012 | Depression Sweet Corn | 0 | 0 | 0 | 0 | 0 | 0 | 0 |
| 1013 | Depression Ornamental/Pop Corn | 0 | 0 | 0 | 0 | 0 | 0 | 0 |
| 1022 | Depression Durum Wheat | 0 | 0 | 0 | 0 | 0 | 0 | 0 |
| 1024 | Depression Winter Wheat | 0 | 0 | 0 | 0 | 0 | 0 | 0 |
| 1027 | Depression Rye | 0 | 0 | 0 | 0 | 0 | 0 | 0 |
| 1028 | Depression Oats | 0 | 0 | 0 | 0 | 0 | 0 | 0 |
| 1036 | Depression Alfalfa | 0 | 0 | 0 | 0 | 0 | 0 | 0 |
| 1037 | Depression Other Hays | 0 | 0 | 0 | 0 | 0 | 0 | 0 |
| 1059 | Depression Seed/Sod Grass | 0 | 0 | 0 | 0 | 0 | 0 | 0 |
| 1061 | Depression Fallow/Idle Cropland | 0 | 0 | 0 | 0 | 0 | 0 | 0 |
| 1111 | Depression NLCD-Open Water | 0 | 0 | 0 | 0 | 0 | 0 | 0 |
| 1121 | Depression NLCD-Open Space | 0 | 0 | 0 | 0 | 0 | 0 | 0 |
| 1122 | Depression NLCD-Developed/Low Intensity | 0 | 0 | 0 | 0 | 0 | 0 | 0 |
| 1123 | Depression NLCD-Developed/Medium Intensity | 0 | 0 | 0 | 0 | 0 | 0 | 0 |
| 1124 | Depression NLCD-Developed/High Intensity | 0 | 0 | 0 | 0 | 0 | 0 | 0 |
| 1131 | Depression NLCD-Barren | 0 | 0 | 0 | 0 | 0 | 0 | 0 |
| 1141 | Depression NLCD-Deciduous Forest | 0 | 0 | 0 | 0 | 0 | 0 | 0 |
| 1142 | Depression NLCD-Evergreen Forest | 0 | 0 | 0 | 0 | 0 | 0 | 0 |
| 1143 | Depression NLCD-Mixed Forest | 0 | 0 | 0 | 0 | 0 | 0 | 0 |
| 1152 | Depression NLCD-Shrubland | 0 | 0 | 0 | 0 | 0 | 0 | 0 |
| 1176 | Depression Grassland/Pasture | 0 | 0 | 0 | 0 | 0 | 0 | 0 |
| 1190 | Depression NLCD-Woody Wetland | 0 | 0 | 0 | 0 | 0 | 0 | 0 |
| 1195 | Depression NLCD-Herbaceous Wetland | 0 | 0 | 0 | 0 | 0 | 0 | 0 |

**Supplemental Table 6.** Integrated Valuation of Ecosystem Services and Tradeoffs (InVEST) threats table for diving ducks in the Iowa portion of the Prairie Pothole Region, USA.

| THREAT | MAX_DIST | WEIGHT | DECAY | BASE_PATH | CUR_PATH | FUT_PATH |
| --- | --- | --- | --- | --- | --- | --- |
| dry | 0.1 | 1 | linear |  | dry_c.tif |  |
| crop | 1 | 1 | linear |  | crop_c.tif |  |
| wetarea | 0.1 | 1 | linear |  | wetarea_c.tif |  |
| roost | 4 | 1 | linear |  | roost_c.tif |  |
| road | 0.5 | 1 | linear |  | road_c.tif |  |

**Supplemental Table 7.** Integrated Valuation of Ecosystem Services and Tradeoffs (InVEST) sensitivity table indicating the relative sensitivity of specific habitat types to specific threats for shorebirds in the Iowa portion of the Prairie Pothole Region, USA. LULC = land use land cover; NLCD = National land cover database. Unsuitable habitat = 0; potentially suitable habitat = 0.5; maximum potentially suitable habitat = 1.

| LULC | NAME | HABITAT | dry | crop | wetarea | roost | road | corn |
| --- | --- | --- | --- | --- | --- | --- | --- | --- |
| 0 | Background | 0 | 0 | 0 | 0 | 0 | 0 | 0 |
| 1 | Corn | 0 | 0 | 0 | 0 | 0 | 0 | 0 |
| 4 | Sorgum | 0 | 0 | 0 | 0 | 0 | 0 | 0 |
| 5 | Soybeans | 0 | 0 | 0 | 0 | 0 | 0 | 0 |
| 6 | Sunflowers | 0 | 0 | 0 | 0 | 0 | 0 | 0 |
| 12 | Sweet Corn | 0 | 0 | 0 | 0 | 0 | 0 | 0 |
| 13 | Ornamental/Pop Corn | 0 | 0 | 0 | 0 | 0 | 0 | 0 |
| 21 | Barley | 0 | 0 | 0 | 0 | 0 | 0 | 0 |
| 22 | Durum Wheat | 0 | 0 | 0 | 0 | 0 | 0 | 0 |
| 23 | Spring Wheat | 0 | 0 | 0 | 0 | 0 | 0 | 0 |
| 24 | Winter Wheat | 0 | 0 | 0 | 0 | 0 | 0 | 0 |
| 25 | Other Small Grains | 0 | 0 | 0 | 0 | 0 | 0 | 0 |
| 26 | Wheat/Soybeans Doubl Crop | 0 | 0 | 0 | 0 | 0 | 0 | 0 |
| 27 | Rye | 0 | 0 | 0 | 0 | 0 | 0 | 0 |
| 28 | Oats | 0 | 0 | 0 | 0 | 0 | 0 | 0 |
| 29 | Millet | 0 | 0 | 0 | 0 | 0 | 0 | 0 |
| 31 | Canola | 0 | 0 | 0 | 0 | 0 | 0 | 0 |
| 32 | Flaxseed | 0 | 0 | 0 | 0 | 0 | 0 | 0 |
| 33 | Safflower | 0 | 0 | 0 | 0 | 0 | 0 | 0 |
| 34 | Rape Seed | 0 | 0 | 0 | 0 | 0 | 0 | 0 |
| 35 | Mustard | 0 | 0 | 0 | 0 | 0 | 0 | 0 |
| 36 | Alfalfa | 0 | 0 | 0 | 0 | 0 | 0 | 0 |
| 37 | Other Hays | 0 | 0 | 0 | 0 | 0 | 0 | 0 |
| 38 | Camelina | 0 | 0 | 0 | 0 | 0 | 0 | 0 |
| 39 | Seed Crop | 0 | 0 | 0 | 0 | 0 | 0 | 0 |
| 41 | Sugarbeets | 0 | 0 | 0 | 0 | 0 | 0 | 0 |
| 42 | Dry Beans | 0 | 0 | 0 | 0 | 0 | 0 | 0 |
| 43 | Potatoes | 0 | 0 | 0 | 0 | 0 | 0 | 0 |
| 44 | Other Crops | 0 | 0 | 0 | 0 | 0 | 0 | 0 |
| 47 | Misc Veg/Fruits | 0 | 0 | 0 | 0 | 0 | 0 | 0 |
| 52 | Lentils | 0 | 0 | 0 | 0 | 0 | 0 | 0 |
| 53 | Peas | 0 | 0 | 0 | 0 | 0 | 0 | 0 |
| 57 | Herbs | 0 | 0 | 0 | 0 | 0 | 0 | 0 |
| 58 | Clover/Wildflowers | 0 | 0 | 0 | 0 | 0 | 0 | 0 |
| 59 | Seed/Sod Grass | 0 | 0 | 0 | 0 | 0 | 0 | 0 |
| 60 | Switchgrass | 0 | 0 | 0 | 0 | 0 | 0 | 0 |
| 61 | Fallow/Idle Cropland | 0 | 0 | 0 | 0 | 0 | 0 | 0 |
| 62 | Pasture/Range/CRP/Non-Ag | 0 | 0 | 0 | 0 | 0 | 0 | 0 |
| 63 | Woodland | 0 | 0 | 0 | 0 | 0 | 0 | 0 |
| 64 | Shrubland | 0 | 0 | 0 | 0 | 0 | 0 | 0 |
| 65 | Barren | 0 | 0 | 0 | 0 | 0 | 0 | 0 |
| 68 | Apples | 0 | 0 | 0 | 0 | 0 | 0 | 0 |
| 69 | Grapes | 0 | 0 | 0 | 0 | 0 | 0 | 0 |
| 70 | Christmas Trees | 0 | 0 | 0 | 0 | 0 | 0 | 0 |
| 82 | Urban/Developed | 0 | 0 | 0 | 0 | 0 | 0 | 0 |
| 83 | Water | 0.5 | 1 | 1 | 0 | 1 | 1 | 0 |
| 87 | Wetlands | 0.5 | 1 | 1 | 0 | 1 | 1 | 0 |
| 88 | Non-ag/Undefined | 0 | 0 | 0 | 0 | 0 | 0 | 0 |
| 111 | NLCD-Open Water | 0.5 | 1 | 1 | 0 | 1 | 1 | 0 |
| 121 | NLCD-Open Space | 0 | 0 | 0 | 0 | 0 | 0 | 0 |
| 122 | NLCD-Developed/Low Intensity | 0 | 0 | 0 | 0 | 0 | 0 | 0 |
| 123 | NLCD-Developed/Medium Intensity | 0 | 0 | 0 | 0 | 0 | 0 | 0 |
| 124 | NLCD-Developed/High Intensity | 0 | 0 | 0 | 0 | 0 | 0 | 0 |
| 131 | NLCD-Barren | 0 | 0 | 0 | 0 | 0 | 0 | 0 |
| 141 | NLCD-Deciduous Forest | 0 | 0 | 0 | 0 | 0 | 0 | 0 |
| 142 | NLCD-Evergreen Forest | 0 | 0 | 0 | 0 | 0 | 0 | 0 |
| 143 | NLCD-Mixed Forest | 0 | 0 | 0 | 0 | 0 | 0 | 0 |
| 152 | NLCD-Shrubland | 0 | 0 | 0 | 0 | 0 | 0 | 0 |
| 171 | NLCD-Grassland Herbaceous | 0 | 0 | 0 | 0 | 0 | 0 | 0 |
| 176 | Grassland/Pasture | 0 | 0 | 0 | 0 | 0 | 0 | 0 |
| 181 | NLCD-Pasture Hayland | 0 | 0 | 0 | 0 | 0 | 0 | 0 |
| 182 | NLCD-Croplands | 0 | 0 | 0 | 0 | 0 | 0 | 0 |
| 190 | NLCD-Woody Wetland | 0 | 0 | 0 | 0 | 0 | 0 | 0 |
| 195 | NLCD-Herbaceous Wetland | 1 | 1 | 1 | 0 | 1 | 1 | 0 |
| 205 | Triticale | 0 | 0 | 0 | 0 | 0 | 0 | 0 |
| 221 | Strawberries | 0 | 0 | 0 | 0 | 0 | 0 | 0 |
| 224 | Vetch | 0 | 0 | 0 | 0 | 0 | 0 | 0 |
| 225 | Dbl. Crop WinWht/Corn | 0 | 0 | 0 | 0 | 0 | 0 | 0 |
| 229 | Pumpkins | 0 | 0 | 0 | 0 | 0 | 0 | 0 |
| 236 | Dbl. Crop WinWht/Sorghum | 0 | 0 | 0 | 0 | 0 | 0 | 0 |
| 240 | Dbl. Crop Soybeans/Oats | 0 | 0 | 0 | 0 | 0 | 0 | 0 |
| 241 | Dbl. Crop Corn/Soybeans | 0 | 0 | 0 | 0 | 0 | 0 | 0 |
| 246 | Radishes | 0 | 0 | 0 | 0 | 0 | 0 | 0 |
| 401 | Temporary Wetland | 1 | 1 | 1 | 0 | 1 | 1 | 0 |
| 402 | Seasonal Wetland | 1 | 1 | 1 | 0 | 1 | 1 | 0 |
| 403 | Semipermanent Wetland | 0.5 | 1 | 1 | 0 | 1 | 1 | 0 |
| 404 | Permanent Wetlands | 0 | 0 | 0 | 0 | 0 | 0 | 0 |
| 405 | Lacustrine | 0 | 0 | 0 | 0 | 0 | 0 | 0 |
| 406 | Riverine Non Intermittent | 0 | 0 | 0 | 0 | 0 | 0 | 0 |
| 407 | Artificially Flooded Wetland | 0.5 | 1 | 1 | 0 | 1 | 1 | 0 |
| 408 | Wetland 160 M Buffer | 0 | 0 | 0 | 0 | 0 | 0 | 0 |
| 411 | WQ wetland | 0 | 0 | 0 | 0 | 0 | 0 | 0 |
| 412 | WQ easement | 0 | 0 | 0 | 0 | 0 | 0 | 0 |
| 413 | WQ Wetland 160m buffer habitat | 0 | 0 | 0 | 0 | 0 | 0 | 0 |
| 600 | Grassland Wetland | 0 | 0 | 0 | 0 | 0 | 0 | 0 |
| 601 | Road | 0 | 0 | 0 | 0 | 0 | 0 | 0 |
| 602 | Lacustrine Perimeter | 0.5 | 1 | 1 | 0 | 1 | 1 | 0 |
| 603 | Riverine Intermittent Streams | 0.5 | 1 | 1 | 0 | 1 | 1 | 0 |
| 604 | WQ Wetland Perimeter | 0.5 | 1 | 1 | 0 | 1 | 1 | 0 |
| 1001 | Depression Corn | 1 | 1 | 1 | 0 | 1 | 1 | 0 |
| 1005 | Depression Soy | 1 | 1 | 1 | 0 | 1 | 1 | 0 |
| 1012 | Depression Sweet Corn | 1 | 1 | 1 | 0 | 1 | 1 | 0 |
| 1013 | Depression Ornamental/Pop Corn | 1 | 1 | 1 | 0 | 1 | 1 | 0 |
| 1022 | Depression Durum Wheat | 1 | 1 | 1 | 1 | 1 | 1 | 0 |
| 1024 | Depression Winter Wheat | 1 | 1 | 1 | 1 | 1 | 1 | 0 |
| 1027 | Depression Rye | 1 | 1 | 1 | 0 | 1 | 1 | 0 |
| 1028 | Depression Oats | 1 | 1 | 1 | 0 | 1 | 1 | 0 |
| 1036 | Depression Alfalfa | 1 | 1 | 1 | 0 | 1 | 1 | 0 |
| 1037 | Depression Other Hays | 1 | 1 | 1 | 0 | 1 | 1 | 0 |
| 1059 | Depression Seed/Sod Grass | 1 | 1 | 1 | 0 | 1 | 1 | 0 |
| 1061 | Depression Fallow/Idle Cropland | 1 | 1 | 1 | 0 | 1 | 1 | 0 |
| 1111 | Depression NLCD-Open Water | 0.5 | 1 | 1 | 0 | 1 | 1 | 0 |
| 1121 | Depression NLCD-Open Space | 1 | 1 | 1 | 0 | 1 | 1 | 0 |
| 1122 | Depression NLCD-Developed/Low Intensity | 0 | 0 | 0 | 0 | 0 | 0 | 0 |
| 1123 | Depression NLCD-Developed/Medium Intensity | 0 | 0 | 0 | 0 | 0 | 0 | 0 |
| 1124 | Depression NLCD-Developed/High Intensity | 0 | 0 | 0 | 0 | 0 | 0 | 0 |
| 1131 | Depression NLCD-Barren | 0.5 | 1 | 1 | 0 | 1 | 1 | 0 |
| 1141 | Depression NLCD-Deciduous Forest | 0 | 0 | 0 | 0 | 0 | 0 | 0 |
| 1142 | Depression NLCD-Evergreen Forest | 0 | 0 | 0 | 0 | 0 | 0 | 0 |
| 1143 | Depression NLCD-Mixed Forest | 0 | 0 | 0 | 0 | 0 | 0 | 0 |
| 1152 | Depression NLCD-Shrubland | 0 | 0 | 0 | 0 | 0 | 0 | 0 |
| 1176 | Depression Grassland/Pasture | 1 | 1 | 1 | 0 | 1 | 1 | 0 |
| 1190 | Depression NLCD-Woody Wetland | 0 | 0 | 0 | 0 | 0 | 0 | 0 |
| 1195 | Depression NLCD-Herbaceous Wetland | 1 | 1 | 1 | 0 | 1 | 1 | 0 |

**Supplemental Table 8.** Integrated Valuation of Ecosystem Services and Tradeoffs (InVEST) threats table for shorebirds in the Iowa portion of the Prairie Pothole Region, USA.

| THREAT | MAX_DIST | WEIGHT | DECAY | BASE_PATH | CUR_PATH | FUT_PATH |
| --- | --- | --- | --- | --- | --- | --- |
| dry | 0.1 | 1 | linear |  | dry_c.tif |  |
| crop | 1 | 1 | linear |  | crop_c.tif |  |
| roost | 10 | 1 | linear |  | roost_c.tif |  |
| road | 0.5 | 1 | linear |  | road_c.tif |  |

**Supplemental Table 9.** Drainage modernization methods results table for waterbirds in the Iowa portion of the Prairie Pothole Region, USA. DWSE = Dynamic Surface Water Extent.

| **Group** | **DSWE Summarization Method** | **Depressional Frequency Threshold** | **Habitat Quality Change from Baseline Due to Drainage Improvement (%)** |
| --- | --- | --- | --- |
| Dabbling Field Foraging Ducks | Presence/Absence | No Depressions Removed | -87% |
|  |  | Depressions with Water < 1 out of 7 Years Removed | -54% |
|  | All Measurements | No Depressions Removed | -87% |
|  |  | Depressions with Water in < 1% of All Measurements Removed | -56% |
| Dabbling Wetland Obligate Ducks | Presence/Absence | No Depressions Removed | -87% |
|  |  | Depressions with Water < 1 out of 7 Years Removed | -54% |
|  | All Measurements | No Depressions Removed | -87% |
|  |  | Depressions with Water in < 1% of All Measurements Removed | -56% |
| Diving Ducks | Presence/Absence | No Depressions Removed | -5% |
|  |  | Depressions with Water < 1 out of 7 Years Removed | -5% |
|  | All Measurements | No Depressions Removed | -5% |
|  |  | Depressions with Water in < 1% of All Measurements Removed | -5% |
| Shorebirds | Presence/Absence | No Depressions Removed | -87% |
|  |  | Depressions with Water < 1 out of 7 Years Removed | -47% |
|  | All Measurements | No Depressions Removed | -87% |
|  |  | Depressions with Water in < 1% of All Measurements Removed | -48% |
