# Supplementary material for: Modeling Wetland Resources for Spring Migratory Waterbirds Under Different Agricultural Management Scenarios in the Iowa Portion of the Prairie Pothole Region, USA
Source: Wetlands (Wilmington). Author manuscript; Available in PMC 2025 Jun 30. (PMC12207345; doi:10.1007/s13157-025-01930-y)
Supplement: Supplementary Material 1 [file NIHMS2086946-supplement-Supplementary_Material_1.docx]

**Supplementary Figures**

**Supplemental Figure 1.** Total Dynamic Surface Water Extent (DSWE) surface water observations for modeled depressional areas across all modeled watersheds in the Iowa portion of the Prairie Pothole Region, USA for the March-April duck observation period.

**Supplemental Figure 2**. Total Dynamic Surface Water Extent (DSWE) surface water observations for modeled depressional areas across all modeled watersheds in the Iowa portion of the Prairie Pothole Region, USA for the April-May shorebird observation period.

**Supplemental Figure 3**. Dynamic Surface Water Extent (DSWE) surface water frequency (%) by depressional area (ha) across all modeled watersheds in the Iowa portion of the Prairie Pothole Region, USA for the March-April observation period. Note breaks in the vertical axis at 5 ha and 150 ha.


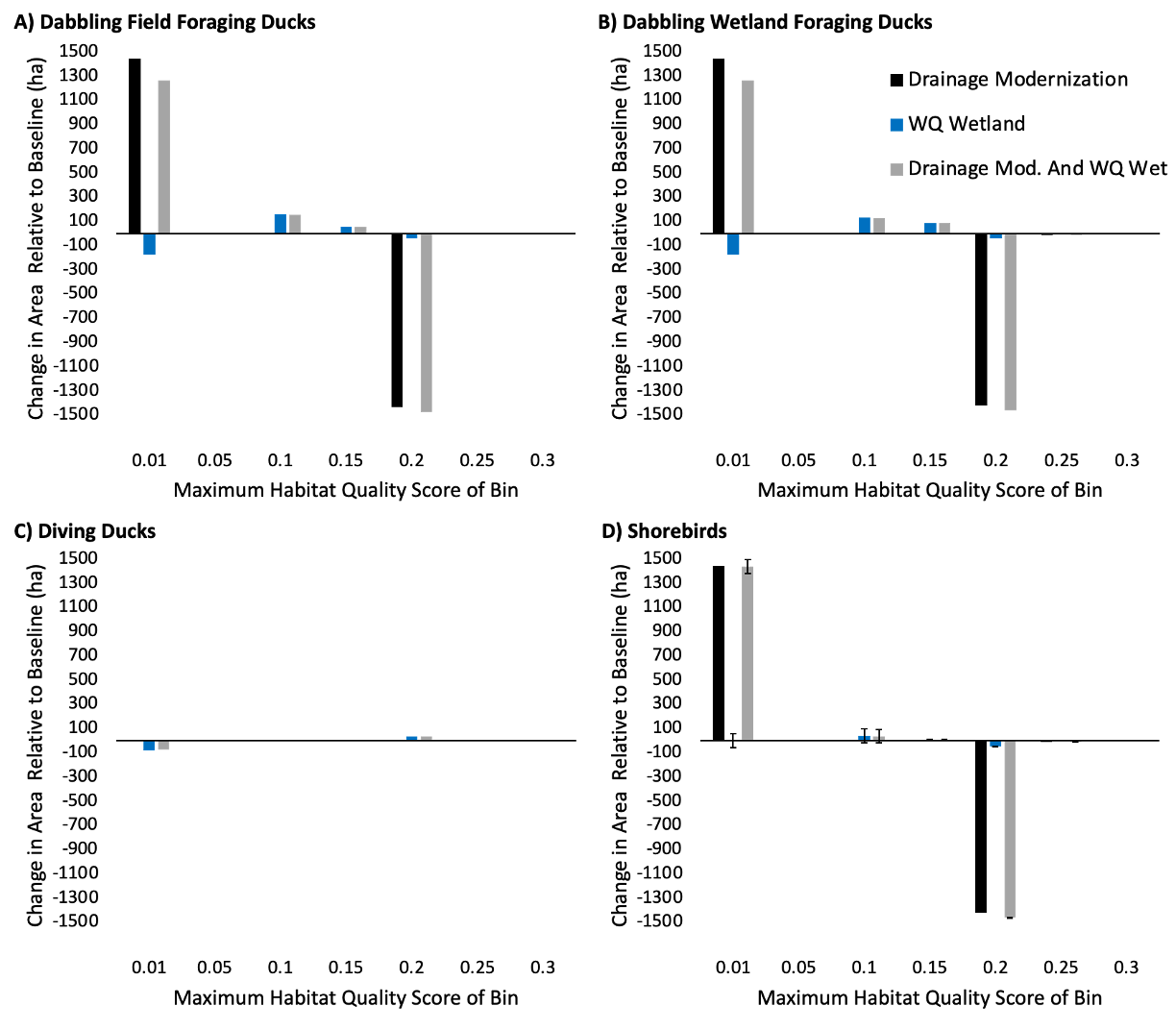


**Supplemental Figure 4.** Change in the area (ha) of habitat quality score ranges (bins) for each scenario (drainage modernization (black bars), water quality wetland additions (blue bars), and drainage modernization combined with water quality wetland additions (gray bars)) relative to the baseline scenario. Results shown for dabbling field foraging ducks (Panel A), dabbling wetland foraging ducks (Panel B), diving ducks (Panel C), and shorebirds (Panel D) in the Iowa portion of the Prairie Pothole Region, USA. Areas correspond to the sum of area for each scenario and each habitat quality score range (bin) across all 37 modeled catchments. For shorebirds in Panel D, error bars correspond to the maximum and minimum areas for the shoreline and no shoreline models in scenarios with water quality wetlands.

**Supplemental Figure 5.** Dynamic Surface Water Extent (DSWE) surface water frequency observations for each modeled depression across all modeled watersheds in the Iowa portion of the Prairie Pothole Region, USA for the April-May observation period. Note breaks in the vertical axis at 6 ha and 150 ha.
